# 2-Amino- and 2-Alkylthio-4*H*-3,1-benzothiazin-4-ones: Synthesis, Interconversion and Enzyme Inhibitory Activities

**DOI:** 10.3390/molecules14010378

**Published:** 2009-01-14

**Authors:** Hans-Georg Häcker, Florian Grundmann, Friederike Lohr, Philipp A. Ottersbach, Jing Zhou, Gregor Schnakenburg, Michael Gütschow

**Affiliations:** 1 Pharmaceutical Institute, Pharmaceutical Chemistry I, University of Bonn, An der Immenburg 4, D-53121 Bonn, Germany; E-mails: hhaecker@uni-bonn.de (H-G. H.), floriangrundmann@gmx.de (F. G.), friederikelohr@gmx.de (F. L.), phil1984@uni-bonn.de (P-A. O.), moshla@uni-bonn.de (J. Z.); 2 Institute of Inorganic Chemistry, University of Bonn, Gerhard-Domagk-Straße 1, D-53121 Bonn, Germany; E-mail: gschnake@uni-bonn.de (G. S.)

**Keywords:** 4*H*-3,1-Benzothiazin-4-ones, Heterocyclisation, Protease inhibition.

## Abstract

The synthetic access to 2-*sec*-amino-4*H*-3,1-benzothiazin-4-ones **2** was explored. Compounds **2** were available from methyl 2-thioureidobenzoates **1**, 2-thioureidobenzoic acids **3**, and novel 2-thioureidobenzamides **6**, respectively, under different conditions. 2-Alkylthio-4*H*-3,1-benzothiazin-4-ones **5** have been prepared from anthranilic acid following a two step route. Both, benzothiazinones **2** and **5** underwent ring cleavage reactions to produce thioureas **1** and **6**, respectively. Twelve benzothiazinones were evaluated as inhibitors against a panel of eight proteases and esterases to identify one selective inhibitor of human cathepsin L, **2b**, and one selective inhibitor of human leukocyte elastase, **5i**.

## Introduction

It was the aim of this study to search for synthetic entries to 4*H*-3,1-benzothiazin-4-ones with amino or alkylthio substituents at position 2. Representatives of this heterocyclic class are assumed to possess biological activities since they might provide four heteroatoms as potential hydrogen bond acceptors and the fused phenyl ring for possible π-π interactions. Analogous 4*H*-3,1-benzoxazin-4-ones have attracted considerable attention as serine hydrolase inhibitors. Their interaction with serine hydrolases involves the acylation of the active-site serine due to enzymatic ring cleavage, followed by slow deacylation of the acyl-enzyme intermediate [1]. 2-Amino and 2-alkylthio substituted 4*H*-3,1-benzoxazin-4-ones have been characterised as potent inhibitors of human leukocyte elastase (HLE) [2,3,4,5], cathepsin G [6, 7], chymase [8], C1r serine protease of the complement system [9, 10], thrombin [11], and human cytomegalovirus protease [12]. 6-Methyl-2-*p*-tolylamino-4*H*-3,1-benzoxazin-4-one (URB754) was identified as a potent inhibitor of the endocannabinoid-deactivating enzyme monoacylglycerol lipase [13]. 2-Aryl substituted 4*H*-3,1-benzoxazin-4-ones have been evaluated as specific inhibitors of the tissue factor/factor VIIa-induced pathway of coagulation [14].

Biological activities of 4*H*-3,1-benzothiazin-4-ones and heterocyclic-fused analogues have been investigated less extensively [15]. Examples include 6-thiaoxanosine, an imidazo[1,5-*a*][1,3]thiazin-7(3*H*)-one riboside with strong antiviral and anticancer properties [16] and the antiproliferative compound 2-(2,4-dihydroxyphenyl)-4*H*-3,1-benzothiazin-4-one [17]. 2-Arylamino substituted thieno[1,3]thiazin-4-ones and analogous [1,3]thiazino[5,4-*b*]indole-4-ones have been reported as inhibitors of HLE [18,19].

We explored the synthetic access to 2-*sec*-amino-4*H*-3,1-benzothiazin-4-ones from different educts. The preparation of 2-alkylthio-4*H*-3,1-benzothiazin-4-ones and their utility to synthesise 2-amino-4*H*-3,1-benzothiazin-4-ones was also investigated. It has been found, that the 2-alkylthio derivatives can indeed serve as precursors for 2-amino analogues in the course of a two-step conversion. The final benzothiazinones were evaluated as inhibitors against a panel of proteases and esterases.

## Results and Discussion

Our initial approach to produce 2-*sec*-amino-4*H*-3,1-benzothiazin-4-ones was the treatment of methyl 2-thioureidobenzoates **1** with concentrated sulphuric acid. This procedure was introduced to prepare 2-aminothieno[2,3-*d*][1,3]thiazin-4-ones [20] and successfully applied to other heterocyclic systems [18, 19, 21,22,23]. Recently, Tarzia *et al.* have prepared the benzothiazine analogue of URB754 that way [24]. Ring closure to 4*H*-3,1-benzothiazine-4-ones was also achieved by treatment of 2-benzoylaminothiobenzamide with concentrated sulphuric acid [25].

**Scheme 1:**
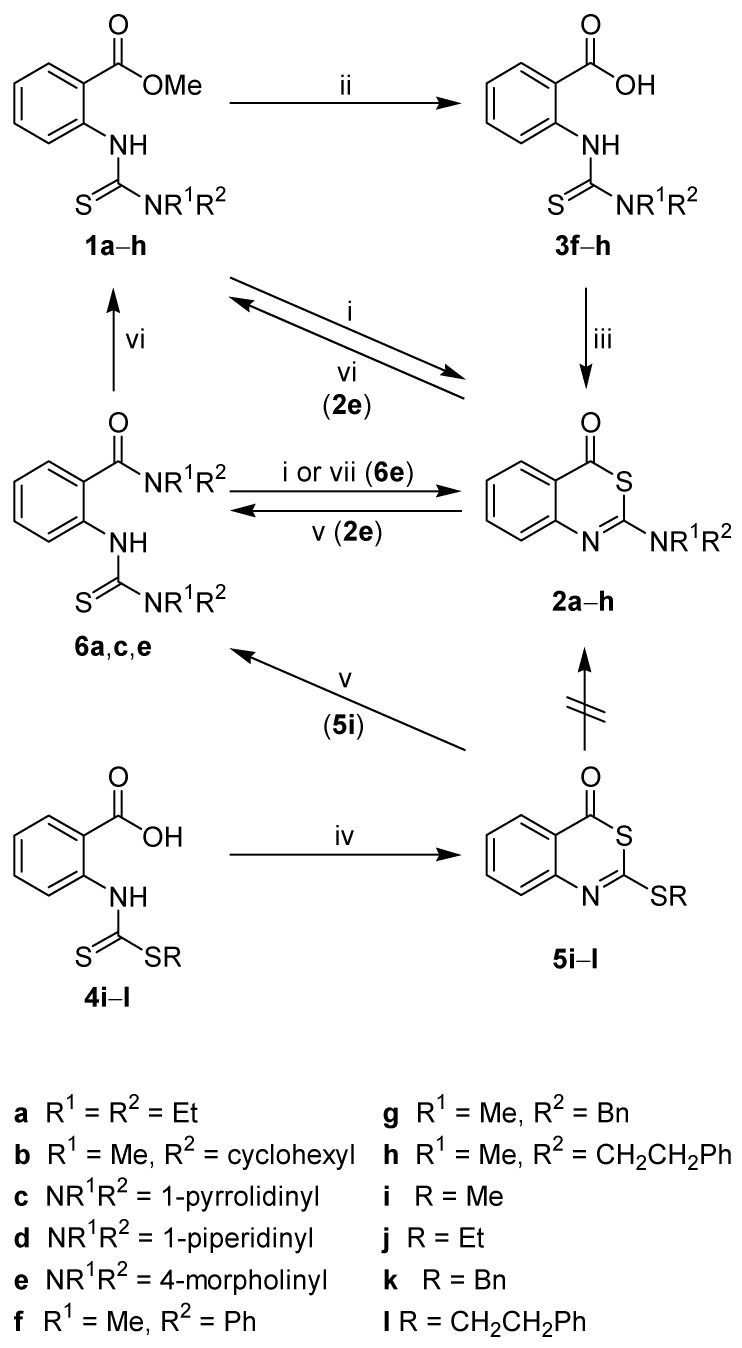
Synthesis and interconversion of 2-amino- and 2-alkylthio-4*H*-3,1-benzothiazin-4-ones.

The new thioureas **1a**–**h** were obtained from methyl 2-isothiocyanatobenzoate and secondary amines (Scheme 1). The treatment of **1a**–**e** with concentrated sulphuric acid at room temperature conveniently afforded the desired benzothiazinones **2a**–**e**. The benzyl(methyl)thiourea derivative **1g** was not converted to **2g** due to *N*-debenzylation under the strong acidic conditions used. The methyl(phenyl)thiourea **1f** gave the corresponding benzothiazinone **2f** in only 20% yield, and the methyl(2-phenylethyl)thiourea **1h** could not be transformed to **2h**. Therefore, an extended synthetic route was chosen. **1f**–**h** were first hydrolyzed to the corresponding benzoic acid derivatives **3f**–**h**, and subsequently cyclised with acetic anhydride [26, 27] to yield **2f**–**h**, thus allowing the facile introduction of aromatic structures within the 2-substituent of **2**. Attempts to directly generate thioureidobenzoic acids **3** from anthranilic acid, 1,1’-thiocarbonyldiimidazole and secondary amines failed (data not shown). 

*Reagents and conditions:* i) concd. H_2_SO_4_, r.t.; ii) NaOH, EtOH, H_2_O, reflux; iii) Ac_2_O, r.t.; iv) Ac_2_O, reflux; v) HNR^1^R^2^, acetone, r.t., then reflux; vi) HCl, MeOH, reflux, 3 h; vii) HCl, MeOH, reflux, 2 min.

A synthetic access to 2-alkylthio-4*H*-3,1-benzothiazin-4-ones was envisaged *via* dithiocarbamates **4i**–**l**, which were prepared from anthranilic acid, carbon disulfide and alkyl halides. These intermediates underwent an easy cyclocondensation upon treatment with acetic anhydride to furnish the new 2-alkylthio derivatives **5i**–**l**. Only one representative of this heterocyclic class, *i.e.* 6,7-difluoro-2-(methylthio)-4*H*-3,1-benzothiazin-4-one, has already been described by Mazuoka *et al*. [28].

**Scheme 2:**
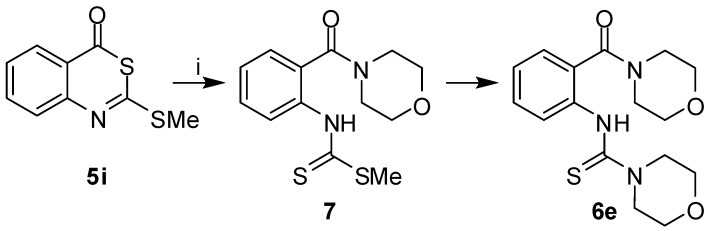
Reaction pathway from **5i** to **6e**.

To explore an alternative entry to 2-*sec*-amino-4*H*-3,1-benzothiazin-4-ones, the *S*-methyl derivative **5i** was reacted with secondary amines. However, 2-aminobenzothiazinones **2** were not formed and instead, we obtained 2-thioureidobenzamides **6a**,**c**,**e**. The attack of an amine on **5i** might either occur at the C-2 or C-4 carbons. An attack at C-2 followed by C-2–S-3 bond breakage would not lead to **6**. The nucleophilic substitution with the release of the methanethiol would generate 2-amino-benzothiazinones **2**. Such intermediates could subsequently undergo ring cleavage due to the attack of the amine at C-4 to produce **6**. When treating the 2-morpholinobenzothiazinone **2e** with morpholine under the conditions used for the conversion of **5i** to **6**, compound **6e** was indeed obtained. However, a different mechanism was proposed based on the isolation of the intermediate **7** in the reaction of **5i** with morpholine (Scheme 2). Hence, the secondary amine attacks the 2-alkylthiobenzothiazinones **5** at C-4, followed by ring opening and subsequent transformation of the dithiocarbamate substituent into a thiourea. Leistner and Wagner reported on a similar formation of 2-thioureido*thio*benzamides when reacting 2-(methylthio)-4*H*-3,1-benzothiazin-4-*thione* with secondary amines [29].

With the novel 2-thioureidobenzamides **6** in hand, we also investigated their utility as precursors to **2**. Indeed, the corresponding 2-aminobenzothiazinones **2a**,**c**,**e** were obtained in quantitative yield and high purity by reacting the benzamide derivatives **6** with concentrated sulphuric acid (Scheme 1).

Heating the 2-thioureidobenzamides **6a**,**c**,**e** in methanolic hydrochloric acid yielded methyl thioureidobenzoates **1a**,**c**,**e**. This transformation is formally an acid-catalyzed amide alcoholysis under conditions where a simple benzamide such as 4-benzoylmorpholine did not react [30]. A ring closure–reopening mechanism operative in the conversion of **6** to **1** is initiated by the rapid cyclocondensation to intermediate 2-aminobenzothiazinones **2**. This could be concluded as the product **2e** was identified after short-time treatment of **6e** with methanolic hydrochloric acid. Prolonged heating of **2e** then led to the formation of the methyl thioureidobenzoate **1e**.

*Reagents and conditions:* i) morpholine, acetone, r.t., 1 h.

**Scheme 3:**
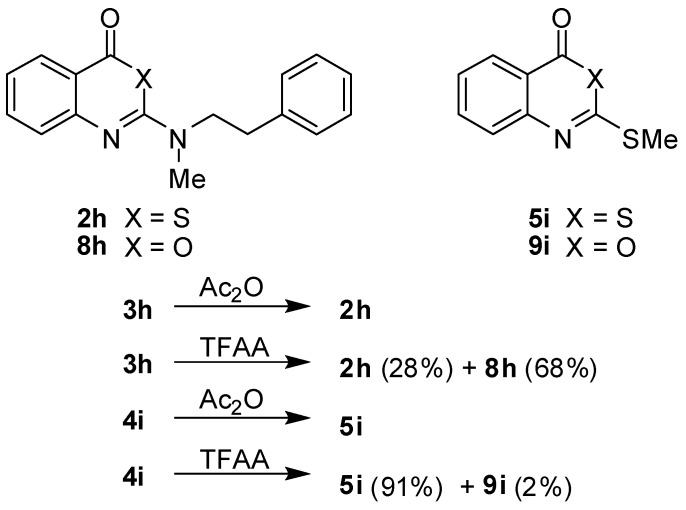
Cyclisation reactions of benzoic acid derivatives **3h** and **4i** with acetic anhydride and trifluoroacetic anhydride.

In the course of this study, acetic anhydride was successfully used in cyclocondensations to convert the benzoic acid derivatives **3** and **4** to benzothiazinones **2** and **5**, respectively. Unexpectedly, the replacement of acetic anhydride by trifluoroacetic anhydride (TFAA) produced different results (Scheme 3). The treatment of **3h** with this reagent gave a mixture of the benzothiazinone **2h** and the benzoxazinone **8h** with the latter compound being the dominant product. On the other hand, the benzothiazinone **5i** was the main product of the reaction of **4i** with TFAA while the corresponding benzoxazinone **9i** was only formed in traces. The formation of **8h** is envisaged to occur by a nucleophilic attack of the carboxyl oxygen at the activated thiocarbonyl carbon [31,32,33,34,35,36]. Further investigations are needed to clarify the mechanism of this desulphurisation-cyclisation. 

**Figure 1:**
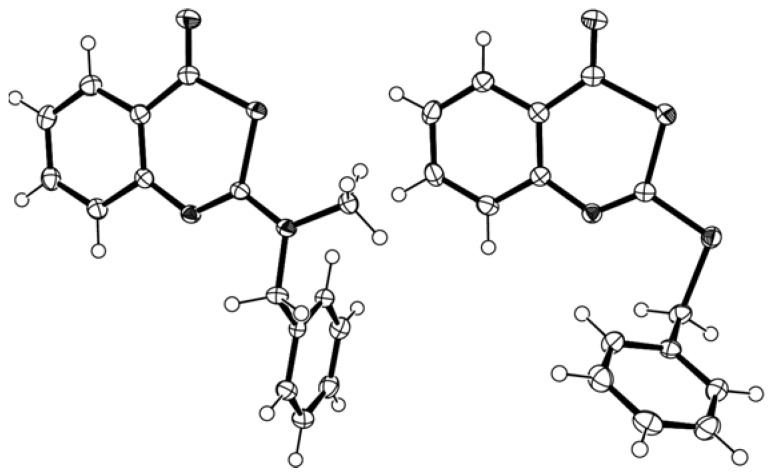
X-ray crystal structure of 2-(*N*-benzyl-*N*-methylamino)-4*H*-3,1-benzothiazin-4-one **2g** (left) and of 2-(benzylthio)-4*H*-3,1-benzothiazin-4-one **5k** (right).

In the ^13^C-NMR spectra of the benzothiazinone representatives **2h** and **5i** the characteristic signals for C-2/C-4 appeared at 156/184 ppm (**2h**) and 164/182 ppm (**5i**). The other benzothiazinones had similar NMR data. The corresponding chemical shifts of the benzoxazinones were observed at 154/160 ppm (**8h**) and 164/159 ppm (**9i**). These values were in accordance with literature data for 4*H*-3,1-benzoxazin-4-ones [14, 32, 37,38,39]. A similar influence of the sulphur-oxygen exchange on the chemical shift of the C-4 carbon was observed for pairs of 2-thien-2-yl and 2-cyano substituted 4*H*-3,1-benzothiazin(oxazin)-4-ones [32, 40]. The structure of the title compounds was furthermore confirmed by X-ray crystal structure analyses [41] (Figure 1). 

The bond lengths within the thiazinone ring of the 2-aminobenzothiazinone **2g** and the 2-alkylthio-benzothiazinone **5k** were similar (see Electronic Supplementary Information). The thiazinone rings adopt an almost planar conformation with the largest deviation from the least square planes defined by the six atoms of the heterocyclic ring being 0.022(1) Å (**2g**) and 0.024(2) Å (**5k**).

2-Aminobenzothiazinones **2a**–**h** and 2-alkylthiobenzothiazinones **5i**–**l** were evaluated as potential inhibitors of HLE [42] (Table 1). Other representative members of serine proteases (human cathepsin G, bovine chymotrypsin and bovine trypsin) were also investigated. The compounds were furthermore assessed towards the cysteine protease human cathepsin L and the metalloprotease angiotensin-converting enzyme (ACE). Two serine esterases, acetylcholinesterase (AChE) and cholesterol esterase (CEase), which share the acyl transfer mechanism with serine proteases were also included in the inhibition studies.

None of the investigated 2-aminobenzothiazinones inhibited HLE. As 2-aminosubstituted 4*H*-3,1-benzoxazin-4-ones are potent inhibitors of HLE, a replacement of the ring oxygen by sulphur resulted in a loss of activity, which can be attributed to the increased intrinsic stability of the benzothiazinones. The second order rate constant for the alkaline hydrolysis of **2e** (1.7 M^-1^s^-1^) was significantly lower than that of the analogous 2-(morpholin-4-yl)-4*H*-3,1-benzoxazin-4-one (28 M^-1^s^-1^) [43]. 2-(*N*-Cyclohexyl-*N*-methylamino)-4*H*-3,1-benzothiazin-4-one (**2b**) exhibited a remarkable inhibitory capacity against human cathepsin L [44]. This compound was selective for cathepsin L with respect to the other enzymes investigated in this study. It might therefore serve as a lead structure for cysteine protease inhibitors. Further investigations are needed to inspect selectivity among cysteine proteases.

**Figure 2:**
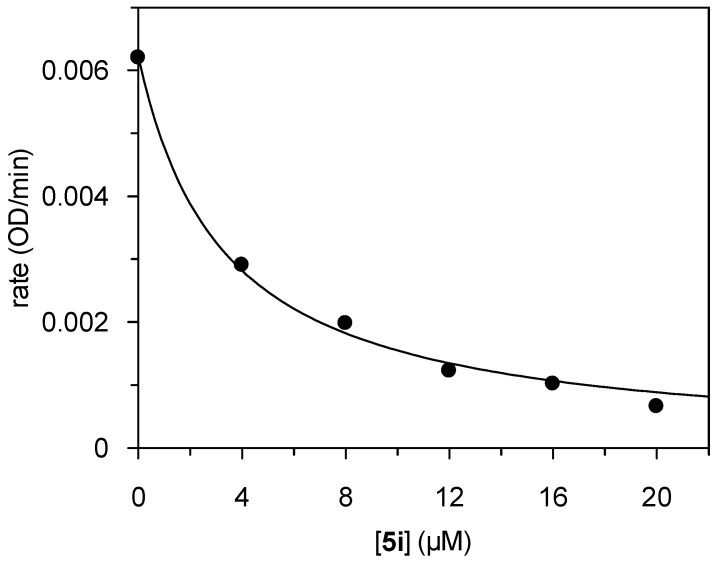
Plot of the steady-state rates *versus* inhibitor concentration for the inhibition of HLE by compound **5i**.

Two of the 2-alkylthiobenzothiazinones were identified as HLE inhibitors. The 2-methylthio and 2-ethylthio derivatives, **5i** and **5j**, exhibited IC_50_ values in the low micromolar range. These compounds carry 2-substituents with the least steric demand among all the benzothiazinones tested. HLE has a primary substrate specificity for small aliphatic amino acid residues at P^1^ position. It can therefore be assumed, that the alkylthio moiety is accommodated by the S^1^ subsite of HLE. The concentration-dependent inhibition by **5i** is presented in Figure 2. The progress curves of the HLE-catalyzed substrate consumption were linear over the 10-min time course. Thus, the time-independent inhibition indicated a non-covalent interaction of **5i** with HLE. Provided that **5i** behaved kinetically as a competitive inhibitor, a *K*_i_ value of 1.2 µM corresponds to the IC_50_ value of 3.3 µM [45]. Noteworthy, the 2-methylthiobenzothiazinone **5i** did not inhibit any of the other enzymes studied here.

**Table 1:** Enzyme inhibitory activities of 2-amino and 2-alkylthio-4*H*-3,1-benzothiazin-4-ones.

				IC_50_ values (µM)^a^				
**Compound**	**HLE**	**Cathepsin G**	**Chymotrypsin**	**Trypsin**	**Cathepsin L**	**ACE**	**AChE**	**CEase**
**2a**	> 100	>100	> 25	> 100	> 50	> 100	>25	> 25
**2b**	> 100	> 50	> 100	> 100	8.93 ± 1.58^b^	> 100	> 50	> 50
**2c**	> 100	> 100	> 100	> 100	> 100	> 100	> 50	> 100
**2d**	> 100	> 100	> 25	> 100	> 50	> 100	> 25	> 50
**2e**	> 100	> 100	> 50	> 100	> 25	> 100	> 25	> 25
**2f**	> 25	> 100	10.4 ± 0.5^c^	> 100	> 50	> 100	> 100	> 50
**2g**	> 25	> 100	22^d^	> 100	22^e^	> 100	> 50	25^f^
**2h**	> 25	> 100	> 50	> 100	> 50	> 100	> 100	> 50
**5i**	3.31 ± 0.24^g^	> 100	> 100	> 100	> 100	> 100	> 50	> 25
**5j**	8.11 ± 0.96^b^	> 100	> 100	> 100	> 100	> 100	> 100	> 25
**5k**	> 25	> 100	18^d^	> 100	> 100	> 100	> 50	> 50
**5l**	> 50	> 100	> 25	> 50	> 100	> 100	19^f^	> 50

^a^ Limits were calculated from duplicate measurements at one or two inhibitor concentrations.

^b^ Triplicate measurement @ five different inhibitor concentrations, see Electronic Supplementary Information.

^c^ Duplicate measurement @ five different inhibitor concentrations, see Electronic Supplementary Information.

^d^ Duplicate measurement @ one inhibitor concentration (10 µM).

^e^ Duplicate measurement @ two inhibitor concentrations (10, 20 µM).

^f^ Quadruplicate measurement @ one inhibitor concentration (5 µM).

^g^ Duplicate measurement @ five different inhibitor concentrations.

## Conclusions

Different routes have been explored to produce 2-*sec*-amino-4*H*-3,1-benzothiazin-4-ones **2**. A particularly versatile method involves the easy saponification of methyl 2-thioureidobenzoates **1** to 2-thioureidobenzoic acids **3**, followed by acetic anhydride-promoted cyclocondensation. The preparation of a series of 2-alkylthio-4*H*-3,1-benzothiazin-4-ones **5** from anthranilic acid using a two step route was demonstrated. We could also show that compounds **5** were cleaved to 2-thioureidobenzamides **6**, which on their own proved to be further precursors to 2-*sec*-amino-4*H*-3,1-benzothiazin-4-ones **2**. Unexpectedly, one 2-aminobenzothiazinone, **2b**, inhibited human cathepsin L, a cysteine protease of therapeutic importance. In the course of this study, biological activities of 2-alkylthio-4*H*-3,1-benzothiazin-4-ones have been evaluated for the first time, and compound **5i** was identified as an inhibitor of human leukocyte elastase.

## Experimental

### General

Solvents and reagents were obtained from Acros (Geel, Belgium), Fluka (Taufkirchen, Germany) or Sigma (Steinheim, Germany), if commercially available. Human leukocyte elastase (HLE), human cathepsin G, human cathepsin L and human angiotensin-converting enzyme (ACE) were obtained from Calbiochem, Darmstadt, Germany. MeOSuc-Ala-Ala-Pro-Val-pNA, Suc-Ala-Ala-Pro-Phe-pNA, Suc-Ala-Ala-Pro-Arg-pNA, Z-Phe-Arg-pNA, and 2-furanacryloyl-phenylalanylglycylglycine (FA-Phe-Gly-Gly) were purchased from Bachem (Bubendorf, Switzerland). Bovine chymotrypsin was purchased from Fluka (Deisenhofen, Germany). Trypsin from bovine pancreas, acetylcholinesterase (AChE) from *Electrophorus electricus*, cholesterol esterase (CEase) from bovine pancreas, 5,5’*-*dithio-bis-(2-nitrobenzoic acid) (DTNB), sodium taurocholate (TC), and *para*-nitrophenylbutyrate (pNPB) were purchased from Sigma (Steinheim, Germany). Methyl 2-isothiocyanatobenzoate was prepared under the conditions reported by Carpenter *et al.* [46]. Thin-layer chromatography was carried out on Merck aluminum sheets, silica gel 60 F_254_. Preparative column chromatography was performed on Merck silica gel 60, 70–230 mesh. Melting points were determined on a Boëtius melting point apparatus (PHMK, VEB Wägetechnik Rapido, Radebeul, Germany) and are uncorrected. ^1^H- and ^13^C-NMR spectra were acquired on a Bruker Avance DRX 500 spectrometer operating at 500 MHz for ^1^H and 125 MHz for ^13^C. Chemical shifts δ are given in ppm referring to the signal center using the solvent peaks for reference: CDCl_3_ 7.26 ppm/77.0 ppm and DMSO-*d*_6_ 2.49 ppm/39.7 ppm. The NMR signals were assigned by two-dimensional ^1^H,^1^H COSY and ^1^H,^13^C correlation spectra (HSQC, HMBC) using standard pulse sequences. Elemental analyses were carried out with a Vario EL apparatus. The spectrophotometric assays were done on Varian Cary 50 Bio and Varian Cary 100 Bio UV/VIS spectrometers with a cell holder equipped with a constant temperature water bath.

### Methyl 2-(3,3-diethylthioureido)benzoate *(**1a**)*

*Method 1:* Diethylamine (0.476 g, 6.5 mmol) was added dropwise to a stirring solution of methyl 2-isothiocyanatobenzoate (0.966 g, 5.0 mmol) in CH_2_Cl_2_ (20 mL). The reaction mixture was stirred at r.t. for 3 h. The organic layer was washed with HCl (0.5 M, 2 × 5 mL), dried over Na_2_SO_4_, filtered, and evaporated to dryness. Recrystallisation from EtOH yielded **1a** (0.946 g, 71%) as colourless needles, mp 85–87 °C (EtOH); ^1^H-NMR (CDCl_3_) δ 1.34 (t, *J =* 6.9 Hz, 6H, CH_2_C*H*_3_), 3.82 (q, *J =* 6.9 Hz, 4H, C*H*_2_CH_3_), 3.88 (s, 3H, CO_2_CH_3_), 7.03 (ddd, *J =* 8.2, 7.3, 1.3 Hz, 1H, 5-H), 7.48 (ddd, *J =* 8.6, 7.3, 1.6 Hz, 1H, 4-H), 7.93 (dd, *J =* 8.2, 1.6 Hz, 1H, 6-H), 8.73 (dd, *J =* 8.6, 1.3 Hz, 1H, 3-H), 10.66 (s, 1H, NH); ^13^C-NMR (CDCl_3_) δ 12.46 (CH_2_*C*H_3_), 45.68 (*C*H_2_CH_3_), 52.31 (CO_2_*C*H_3_), 116.86 (C-1), 122.22 (C-5), 123.62 (C-3), 130.22 (C-6), 132.82 (C-4), 143.28 (C-2), 168.99 (*C*O_2_CH_3_), 179.23 (NHCS); Anal. calcd. for C_13_H_18_N_2_O_2_S: C, 58.6; H, 6.8; N, 10.5. Found: C, 58.4; H, 6.8; N, 10.4.

*Method 2:* 2-(3,3-Diethylthioureido)-*N*,*N*-diethylbenzamide (**6a**, 0.307 g, 1.0 mmol) was heated to reflux in anhydrous methanolic HCl (0.25 M, 5 mL) for 3 h. The mixture was allowed to cool to r.t. and kept at -15 °C. The precipitate was removed by suction filtration to give **1a** (0.169 g, 63%) as white needles.

### Methyl 2-(3-cyclohexyl-3-methylthioureido)benzoate *(**1b**)*

According to the preparation of **1a** (Method 1), **1b** (1.50 g, 98%) was obtained from methyl 2-isothiocyanatobenzoate and *N*-methylcyclohexylamine as a semisolid crude material. ^1^H-NMR (CDCl_3_) δ 1.05–1.95 (m, 10H, 2’/3’/4’/5’/6’-H), 3.20 (s, 3H, NCH_3_), 3.88 (s, 3H, CO_2_CH_3_), 7.03 (ddd, *J =* 8.2, 7.1, 1.3 Hz, 1H, 5-H), 7.48 (ddd, *J =* 8.8, 7.1, 1.6 Hz, 1H, 4-H), 7.93 (dd, *J =* 8.2, 1.6 Hz, 1H, 6-H), 8.71(d, *J =* 8.2 Hz, 1H, 3-H), 10.70 (s, 1H, NH); ^13^C-NMR (CDCl_3_) δ 25.48 (C-4’), 25.54 (C-3’/5’), 30.01 (C-2’/6’), 32.57 (NCH_3_), 52.29 (CO_2_*C*H_3_), 59.23 (C-1’), 116.74 (C-1), 122.16 (C-5), 123.35 (C-3), 130.23 (C-6), 132.86 (C-4), 143.27 (C-2), 168.99 (*C*O_2_CH_3_), 179.90 (NHCS); Anal. calcd. for C_16_H_22_N_2_O_2_S: C, 62.7; H, 7.2; N, 9.1. Found: C, 62.7; H, 6.7; N, 9.0.

### Methyl 2-[(1-pyrrolidinylthiocarbonyl)amino]benzoate *(**1c**)*

*Method 1:* According to the preparation of **1a** (Method 1), **1c** (1.32 g, 82%) was obtained from methyl 2-isothiocyanatobenzoate and pyrrolidine as colourless needles, mp 124–127 °C (EtOH); ^1^H-NMR (CDCl_3_) δ 1.87–2.16 (m, 4H, 3’/4’-H), 3.65–3.96 (m, 7H, CO_2_CH_3_, 2’/5’-H), 7.04 (ddd, *J =* 7.9, 7.3, 1.3 Hz, 1H, 5-H), 7.51 (ddd *J =* 8.5, 7.3, 1.6 Hz, 1H, 4-H), 7.95 (dd, *J =* 7.9, 1.6 Hz, 1H, 6-H), 9.00 (dd, *J =* 8.5, 1.0 Hz, 1H, 3-H), 10.82 (s, 1H, NH); ^13^C-NMR (CDCl_3_) δ 24.59, 26.20 (C-3’/4’), 48.30, 52.13 (C-2’/5’), 52.33 (CO_2_*C*H_3_), 116.40 (C-1), 122.26, 122.65 (C-3/5), 130.35 (C-6), 133.16 (C-4), 142.88 (C-2), 169.05 (*C*O_2_CH_3_), 176.47 (NHCS); Anal. calcd. for C_13_H_16_N_2_O_2_S: C, 59.1; H, 6.1; N, 10.6. Found: C, 59.1; H, 6.35; N, 10.5.

*Method 2:* According to the preparation of **1a **(Method 2), **1c** (0.222 g, 84%) was obtained from **6c** as colourless needles.

### Methyl 2-[(1-piperidinylthiocarbonyl)amino]benzoate *(**1d**)*

According to the preparation of **1a** (Method 1), **1d** (1.11 g, 80%) was obtained from methyl 2-isothiocyanatobenzoate and piperidine as colourless plates, mp 116–117 °C (EtOH); ^1^H-NMR (CDCl_3_) δ 1.68–1.73 (m, 6H, 3’/4’/5’-H), 3.88 (s, 3H, CO_2_CH_3_), 3.95–4.01 (m, 4H, 2’/6’-H), 7.02 (ddd, *J =* 8.2, 7.3, 1.3 Hz, 1H, 5-H), 7.48 (ddd, *J =* 8.8, 7.3, 1.6 Hz, 1H, 4-H), 7.93 (dd, *J =* 8.1, 1.8 Hz, 1H, 6-H), 8.53 (dd, *J =* 8.5, 1.3 Hz, 1H, 3-H), 10.75 (s, 1H, NH); ^13^C-NMR (CDCl_3_) δ 24.40 (C-4’), 25.67 (C-3’/5’), 49.68 (C-2’/6’), 52.33 (CO_2_*C*H_3_), 116.41 (C-1), 122.04 (C-5), 123.03 (C-3), 130.33 (C-6), 132.97 (C-4), 143.40 (C-2), 169.06 (*C*O_2_CH_3_), 179.60(NHCS); Anal. calcd. for C_14_H_18_N_2_O_2_S: C, 60.4; H, 6.5; N, 10.1. Found: C, 60.6; H, 6.55; N, 10.1.

### Methyl 2-[(4-morpholinylthiocarbonyl)amino]benzoate *(**1e**)*

*Method 1:* According to the preparation of **1a**, **1e** (1.11 g, 80%) was obtained from methyl 2-isothiocyanatobenzoate and morpholine as a white solid, mp 103–107 °C (EtOH), lit. [47] 106–110 °C); ^1^H-NMR (CDCl_3_) δ 3.79 (t, *J =* 4.9 Hz, 4H, 2’/6’-H), 3.89 (s, 3H, CO_2_CH_3_), 4.04 (t, *J =* 4.9 Hz, 4H, 3’/5’-H), 7.06 (ddd, *J =* 8.2, 6.9, 1.3 Hz, 1H, 5-H), 7.51 (ddd, *J =* 8.6, 7.3, 1.6 Hz, 1H, 4-H), 7.96 (dd, *J =* 8.1, 1.6 Hz, 1H, 6-H), 8.67 (dd, *J =* 8.5, 1.0 Hz, 1H, 3-H), 10.97 (s, 1H, NH); ^13^C-NMR (CDCl_3_) δ 48.25 (C-3’/5’), 52.48 (CO_2_*C*H_3_), 66.29 (C-2’/6’), 116.57 (C-1), 122.58, 122.93 (C-3/5), 130.43 (C-6), 133.19 (C-4), 142.95 (C-2), 169.19 (*C*O_2_CH_3_), 180.72 (NHCS); Anal. calcd. for C_13_H_16_N_2_O_3_S: C, 55.7; H, 5.75; N, 10.0. Found: C, 56.0; H, 5.9; N, 9.8.

*Method 2:* According to the preparation of **1a** (Method 2), **1e** (0.229 g, 82%) was obtained from **6e** as a light yellow solid.

*Method 3:* 2-(Morpholin-4-yl)-4*H*-3,1-benzothiazin-4-one (**2e**, 0.160 g, 0.64 mmol) was heated to reflux in anhydrous methanolic HCl (0.25 M, 3 mL) for 3 h. The mixture was allowed to cool to r.t. and kept at -15 °C. The precipitate was removed by suction filtration to give **1e** (0.151 g, 84%) as a light yellow solid.

### Methyl 2-(3-methyl-3-phenylthioureido)benzoate *(**1f**)*

According to the preparation of **1a** (Method 1), **1f** (1.28 g, 84%) was obtained from methyl 2-isothiocyanatobenzoate and *N*-methylaniline as colourless needles, mp 70–71 °C (EtOH); ^1^H-NMR (DMSO-*d*_6_) δ 3.61 (s, 3H, NCH_3_), 3.70 (s, 3H, CO_2_CH_3_), 7.15–7.17 (m, 1H, 5-H), 7.39–7.45 (m, 3H, 2’/4’/6’-H), 7.50–7.55 (m, 3H, 4/3’/5’-H), 7.77 (dd, *J =* 8.2, 1.6 Hz, 1H, 6-H), 8.24 (dd, *J =* 8.4, 1.0 Hz, 1H, 3-H), 9.67 (s, 1H, NH); ^13^C-NMR (DMSO-*d*_6_) δ 43.28 (NCH_3_), 52.44 (CO_2_*C*H_3_), 120.31 (C-1), 123.65 (C-5), 125.06 (C-3), 127.01 (C-2’/6’), 128.27 (C-4’), 130.01 (C-6), 130.26 (C-3’/5’), 132.46 (C-4), 141.32 (C-2), 143.71 (C-1’), 167.08 (*C*O_2_CH_3_), 180.48 (NHCS); Anal. calcd. for C_16_H_16_N_2_O_2_S: C, 64.0; H, 5.4; N, 9.3. Found: C, 63.7; H, 5.4; N, 9.3.

### Methyl 2-(3-benzyl-3-methylthioureido)benzoate *(**1g**)*

According to the preparation of **1a** (Method 1), **1g** (1.45 g, 92%) was obtained from methyl 2-isothiocyanatobenzoate and *N*-benzylmethylamine as white plates, mp 88–92 °C (EtOH); ^1^H-NMR (CDCl_3_) δ 3.30 (s, 3H, NCH_3_), 3.87 (s, 3H, CO_2_CH_3_), 5.25 (s, 2H, C*H*_2_Ph), 7.08 (ddd, *J =* 8.4, 7.1, 1.3 Hz, 1H, 5-H), 7.26–7.28 (m, 1H, 4’-H), 7.29–7.34 (m, 4H, 2’/3’/5’/6’-H), 7.53 (ddd, *J =* 8.5, 7.3, 1.6 Hz, 1H, 4-H), 7.96 (dd, *J =* 7.9, 1.6 Hz, 1H, 6-H), 8.87 (d, *J =* 8.6 Hz, 1H, 3-H), 10.93 (s, 1H, NH); ^13^C-NMR (CDCl_3_) δ 37.63 (NCH_3_), 52.36 (CO_2_*C*H_3_), 56.78 (*C*H_2_Ph), 117.06 (C-1), 122.64 (C-5), 123.42 (C-3), 127.52 (C-2’/6’), 127.58 (C-4’), 128.72 (C-3’/5’), 130.31 (C-6), 133.01 (C-4), 136.43 (C-1’), 142.98 (C-2), 168.95 (*C*O_2_CH_3_), 180.94 (NHCS); Anal. calcd. for C_17_H_18_N_2_O_2_S: C, 64.9; H, 5.8; N, 8.9. Found: C, 64.9; H, 6.05; N, 8.9.

### Methyl 2-[3-methyl-3-(2-phenylethyl)thioureido]benzoate *(**1h**)*

According to the preparation of **1a** (Method 1), **1h** (1.47 g, 90%) was obtained from methyl 2-isothiocyanatobenzoate and *N*-methyl phenethylamine as a semisolid crude material; ^1^H-NMR (CDCl_3_) δ 3.07 (t, *J =* 7.9 Hz, 2H, CH_2_C*H*_2_Ph), 3.28 (s, 3H, NCH_3_), 3.90 (s, 3H, CO_2_CH_3_), 4.05–4.14 (m, 2H, C*H*_2_CH_2_Ph), 7.06 (ddd, *J =* 8.2, 6.9, 1.3 Hz, 1H, 5-H), 7.19–7.23 (m, 1H, 4’-H), 7.27–7.31 (m, 4H, 2’/3’/5’/6’-H), 7.51 (ddd, *J =* 8.6, 7.2, 1.6 Hz, 1H, 4-H), 7.96 (dd, *J =* 7.9, 1.6 Hz, 1H, 6-H), 8.80 (d, *J =* 8.6 Hz, 1H, 3-H), 10.84 (s, 1H, NH); ^13^C-NMR (CDCl_3_) δ 33.38 (CH_2_*C*H_2_Ph), 39.22 (NCH_3_), 52.35 (CO_2_*C*H_3_), 55.82 (*C*H_2_CH_2_Ph), 116.80 (C-1) 122.41 (C-5), 123.27 (C-3), 126.52 (C-4’), 128.60 (C-2’/6’), 128.91 (C-3’/5’), 130.27 (C-6), 132.99 (C-4), 138.55 (C-1’), 143.05 (C-2), 169.02 (*C*O_2_CH_3_), 179.87 (NHCS); Anal. calcd. for C_18_H_20_N_2_O_2_S: C, 65.8; H, 6.1; N, 8.5. Found: C, 64.9; H, 5.8; N, 8.9.

### 2-(Diethylamino)-4*H*-3,1-benzothiazin-4-one *(**2a**)*

*Method 1:* Methyl 2-(3,3-diethylthioureido)benzoate (**1a**, 0.799 g, 3.0 mmol) was kept in concd. H_2_SO_4_ (12 mL) at r.t. for 24 h. The solution was poored into a mixture of ice–water (100 mL) and EtOAc (100 mL). After neutralization, the aqueous layer was further extracted with EtOAc (2 × 100 mL). The combined organic layers were dried over Na_2_SO_4_, filtered, and evaporated to dryness. Recrystallisation from MeOH yielded **2a** (0.505 g, 72%) as colourless needles, mp 74–75 °C (MeOH), lit. [43] 72–74 °C; ^1^H-NMR (CDCl_3_) δ 1.24 (t, *J =* 7.3 Hz, 6H, CH_2_C*H*_3_), 3.59 (q, *J =* 7.3 Hz, 4H, C*H*_2_CH_3_), 7.10 (ddd, *J =* 8.2, 7.6, 1.3 Hz, 1H, 6-H), 7.37 (dd, *J =* 7.9, 1.3 Hz, 1H, 8-H), 7.55 (ddd, *J =* 8.5, 6.9, 1.6 Hz, 1H, 7-H), 8.00 (dd, *J =* 8.0, 1.6 Hz, 1H, 5-H); ^13^C-NMR (CDCl_3_) δ 13.03 (CH_2_*C*H_3_), 43.35 (*C*H_2_CH_3_), 116.29 (C-4a), 122.93 (C-6), 124.71 (C-5), 128.21 (C-8), 135.61 (C-7), 151.50 (C-8a), 155.43 (C-2), 184.52 (C-4); Anal. calcd. for C_12_H_14_N_2_OS: C, 61.5; H, 6.0; N, 12.0. Found: C, 61.5; H, 6.0; N, 12.0.

*Method 2:* 2-(3,3-Diethylthioureido)-*N*,*N*-diethylbenzamide **6a** (0.615 g, 2.0 mmol) was treated with concd. H_2_SO_4_ (8 mL) as described under Method 1 obtaining **2a** (0.449 g, 96%) as a white solid.

### 2-(*N*-Cyclohexyl-*N*-methylamino)-4*H*-3,1-benzothiazin-4-one *(**2b**)*

According to the preparation of **2a** (Method 1), **2b** (0.607 g, 74%) was obtained from **1b** as colourless plates, mp 111–114 °C (EtOH); ^1^H-NMR (CDCl_3_) δ 1.05–1.88 (m, 10H, 2’/3’/4’/5’/6’-H), 3.05 (s, 3H, NCH_3_), 4.22 (br s, 1H, 1’-H), 7.10 (ddd, *J =* 8.2, 6.9, 1.3 Hz, 1H, 6-H), 7.38 (dd, *J =* 8.2, 1.3 Hz, 1H, 8-H), 7.56 (ddd, *J =* 8.5, 6.9, 1.6 Hz, 1H, 7-H), 8.00 (dd, *J =* 8.2, 1.6 Hz, 1H, 5-H); ^13^C-NMR (CDCl_3_) δ 25.40 (C-4’), 25.73 (C-3’/5’), 30.14 (C-2’/6’), 30.45 (NCH_3_), 56.82 (C-1’), 116.50 (C-4a), 123.01 (C-6), 124.75 (C-5), 128.18 (C-8), 135.64 (C-7), 151.36 (C-8a), 156.68 (C-2), 184.48 (C-4); Anal. calcd. for C_15_H_18_N_2_OS: C, 65.7; H, 6.6; N, 10.2. Found: C, 65.4; H, 6.6; N, 10.1.

### 2-(Pyrrolidin-1-yl)-4*H*-3,1-benzothiazin-4-one *(**2c**)*

*Method 1:* According to the preparation of **2a** (Method 1), **2c** (0.514 g, 74%) was obtained from **1c** as white needles, mp 105–107 °C (EtOH); ^1^H-NMR (CDCl_3_) δ 1.97–2.04 (m, 4H, 3’/4’-H), 3.58 (br s, 4H, 2’/5’-H), 7.10 (ddd, *J =* 8.2, 6.9, 1.3 Hz, 1H, 6-H), 7.39 (dd, *J =* 8.2, 1.0 Hz, 1H, 8-H), 7.56 (ddd, *J =* 8.5, 7.3, 1.6 Hz, 1H, 7-H), 8.01 (dd, *J =* 8.4, 1.6 Hz, 1H, 5-H); ^13^C-NMR (CDCl_3_) δ 24.97 (C-3’/4’), 47.67 (C-2’/5’), 116.58 (C-4a), 122.87 (C-6), 124.86 (C-5), 128.06 (C-8), 135.65 (C-7), 151.57 (C-8a), 154.62 (C-2), 184.22 (C-4); Anal. calcd. for C_12_H_12_N_2_OS: C, 62.0; H, 5.2; N, 12.1. Found: C, 61.9; H, 5.3; N, 11.9.

*Method 2:* According to the preparation of **2a** (Method 2), **2c** (0.435 g, 94%) was obtained from **6c** as a light yellow solid.

### 2-(Piperidin-1-yl)-4*H*-3,1-benzothiazin-4-one *(**2d**)*

According to the preparation of **2a** (Method 1), **2d** (0.594 g, 80%) was obtained from **1d** as white needles, mp 87–88 °C (EtOH); ^1^H-NMR (CDCl_3_) δ 1.60–1.72 (m, 6H, 3’/4’/5’-H), 3.67–3.72 (m, 4H, 2’/6’-H), 7.12 (ddd, *J =* 8.2, 6.9, 1.3 Hz, 1H, 6-H), 7.36 (dd, *J =* 8.2, 1.0 Hz, 1H, 8-H), 7.56 (ddd, *J =* 8.5, 6.9, 1.6 Hz, 1H, 7-H), 8.00 (dd, *J =* 7.9, 1.6 Hz, 1H, 5-H); ^13^C-NMR (CDCl_3_) δ 24.64 (C-4’), 25.62 (C-3’/5’), 46.84 (C-2’/6’), 116.36 (C-4a), 123.28 (C-6), 124.81 (C-5), 128.13 (C-8), 135.69 (C-7), 151.22 (C-8a), 156.27 (C-2), 184.29 (C-4); Anal. calcd. for C_13_H_14_N_2_OS: C, 63.4; H, 5.7; N, 11.4. Found: C, 63.35; H, 5.9; N, 11.3.

### 2-(Morpholin-4-yl)-4*H*-3,1-benzothiazin-4-one *(**2e**)*

*Method 1:* According to the preparation of **2a** (Method 1), **2e** (0.395 g, 53%) was obtained from **1e** as colourless needles, mp 137–138 °C (EtOH), lit. [48] 136–137 °C; ^1^H-NMR (CDCl_3_) δ 3.71–3.79 (m, 8H, 2’/3’/5’/6’-H), 7.18 (ddd, *J =* 8.0, 6.9, 1.3 Hz, 1H, 6-H), 7.38 (dd, *J =* 8.2, 1.3 Hz, 1H, 8-H), 7.60 (ddd, *J =* 8.5, 6.9, 1.6 Hz, 1H, 7-H), 8.02 (dd, *J =* 8.1, 1.7 Hz, 1H, 5-H); ^13^C-NMR (CDCl_3_) δ 45.90 (C-3’/5’), 66.40 (C-2’/6’), 116.74 (C-4a), 124.08 (C-6), 124.95 (C-5), 128.32 (C-8), 135.88 (C-7), 150.45 (C-8a), 156.75 (C-2), 183.44 (C-4); Anal. calcd. for C_12_H_12_N_2_O_2_S: C, 58.05; H, 4.9; N, 11.3. Found: C, 58.1; H, 4.9; N, 11.2.

*Method 2:* According to the preparation of **2a** (Method 2), **2e** (0.453 g, 92%) was obtained from **6e** as light yellow needles.

*Method 3:* *N*-[2-(Morpholin-4-ylcarbonyl)phenyl]morpholine-4-carbothioamide (**6e**, 0.711 g, 2.0 mmol) was heated under reflux in anhydrous methanolic HCl (0.25 M, 10 mL) for 2 min. After cooling to r.t., the precipitate was removed by suction filtration, washed with H_2_O (30 mL), dried under vacuo to give **2e** (0.380 g, 77%) as light yellow needles.

### 2-(*N*-Methyl-*N*-phenylamino)-4*H*-3,1-benzothiazin-4-one *(**2f**)*

*Method 1:* According to the preparation of **2a** (Method 1), **2f** (0.160 g, 20%) was obtained from **1f** as colourless needles, mp 78–79 °C (EtOH); ^1^H-NMR (CDCl_3_) δ 3.59 (s, 3H, NCH_3_), 7.18 (ddd, *J =* 8.2, 6.9, 1.3 Hz, 1H, 6-H), 7.25–7.29 (m, 2H, 2’/6’-H), 7.37–7.42 (m, 1H, 4’-H), 7.42–7.47 (m, 2H, 3’/5’-H), 7.51 (dd, *J =* 8.2, 1.0 Hz, 1H, 8-H), 7.62 (ddd, *J =* 8.5, 6.9, 1.6 Hz, 1H, 7-H), 8.02 (dd, *J =* 8.0, 1.6 Hz, 1H, 5-H); ^13^C-NMR (CDCl_3_) δ 39.93 (NCH_3_), 117.07 (C-4a), 123.93 (C-6), 125.00 (C-5), 128.26 (C-2’/6’), 128.29 (C-8), 128.76 (C-4’), 130.16 (C-3’/5’), 135.72 (C-7), 142.20 (C-1’), 150.50 (C-8a), 156.97 (C-2), 184.20 (C-4); Anal. calcd. for C_15_H_12_N_2_OS: C, 67.1; H, 4.5; N, 10.4. Found: C, 67.0; H, 4.6; N, 10.4.

*Method 2*: 2-(3-Methyl-3-phenylthioureido)benzoic acid (**3f**, 0.859 g, 3.0 mmol) and Ac_2_O (7.0 mL) were kept at r.t. for 12 h. The solvent was removed under reduced pressure. Recrystallisation from EtOH gave **2f** (0.346 g, 43%).

### 2-(*N*-Benzyl-*N*-methylamino)-4*H*-3,1-benzothiazin-4-one *(**2g**)*

2-(3-Benzyl-3-methylthioureido)benzoic acid (**3g**, 0.150 g, 0.50 mmol) and Ac_2_O (1.0 mL) were kept at r.t. for 8 h. The resulting crystals were removed by suction filtration to obtain **2g** (0.109 g, 77%) as colourless needles, mp 70–71 °C; ^1^H-NMR (CDCl_3_) δ 3.13 (s, 3H, NCH_3_), 4.87 (s, 2H, C*H*_2_Ph), 7.15 (ddd, *J =* 8.0, 6.9, 1.0 Hz, 1H, H-6), 7.26–7.36 (m, 5H, H-2’/3’/4’/5’/6’), 7.42 (dd, *J =* 8.2, 1.0 Hz, 1H, H-8), 7.59 (ddd, *J =* 8.4, 6.9, 1.6 Hz, 1H, H-7), 8.03 (dd, *J =* 8.0, 1.6 Hz, 1H, H-5); ^13^C-NMR (CDCl_3_) δ 35.95 (NCH_3_), 53.56 (*C*H_2_Ph), 116.34 (C-4a), 123.42 (C-6), 124.84 (C-5), 127.58 (C-2’/6’), 127.73 (C-4’), 128.30 (C-8), 128.79 (C-3’/5’), 135.76 (C-7), 136.31 (C-1’), 151.04 (C-8a), 157.07 (C-2), 183.95 (C-4); Anal. calcd. for C_16_H_14_N_2_OS: C, 68.1; H, 5.0; N, 9.9. Found: C, 67.7; H, 5.2; N, 9.8.

### 2-[*N*-Methyl-*N*-(2-phenylethyl)amino]-4*H*-3,1-benzothiazin-4-one *(**2h**)*

*Method 1:* According to the preparation of **2f** (Method 2), **2h** (0.578 g, 65%) was obtained from **3h** as a white solid, mp 72–75 °C (EtOH); ^1^H-NMR (CDCl_3_) δ 2.96 (t, *J =* 7.6 Hz, 2H, CH_2_C*H*_2_Ph), 3.08 (s, 3H, NCH_3_), 3.81 (t, *J =* 7.6 Hz, 2H, C*H*_2_CH_2_Ph), 7.14 (ddd, *J =* 8.2, 7.1, 1.3 Hz, 1H, 6-H), 7.20–7.33 (m, 5H, 2’/3’/4’/5’/6’-H), 7.44 (dd, *J =* 8.2, 1.0 Hz, 1H, 8-H), 7.59 (ddd, *J =* 8.2, 6.9, 1.6 Hz, 1H, 7-H), 8.03 (dd, *J =* 7.9, 1.6 Hz, 1H, 5-H); ^13^C-NMR (CDCl_3_) δ 33.76 (CH_2_*C*H_2_Ph), 37.07 (NCH_3_), 53.03 (*C*H_2_CH_2_Ph), 116.23 (C-4a), 123.38 (C-6), 124.83 (C-5), 126.68 (C-4’), 128.18 (C-8), 128.70 (C-2’/6’), 128.84 (C-3’/5’), 135.77 (C-7), 138.32 (C-1’), 150.90 (C-8a), 156.35 (C-2), 183.91 (C-4); Anal. calcd. for C_17_H_16_N_2_OS: C, 68.9; H, 5.4; N, 9.45. Found: C, 68.9; H, 5.4; N, 9.5.

*Method 2:* 2-[3-Methyl-3-(2-phenylethyl)thioureido]benzoic acid (**3h**, 0.940 g, 3.0 mmol) and TFAA (7.0 mL) were kept at r.t. for 12 h. After removal of the solvent, the resulting crude material was purified by column chromatography on silica using petroleum ether/EtOAc (8+1) as eluent to give **2h** (0.249 g, 28%) as a yellowish solid.

### 2-(3-Methyl-3-phenylthioureido)benzoic acid *(**3f**)*

A mixture of methyl 2-(3-methyl-3-phenylthioureido)benzoate (**1f**, 0.601 g, 2.0 mmol), aqueous NaOH (1 M, 10 mL) and EtOH (10 mL) was heated to reflux for 1 h. The reaction was allowed to cool to r.t. and H_2_O (30 mL) was added. After filtration and cooling to 0 °C, the solution was slowly acidified with concd. HCl. The precipitate was removed by suction filtration and washed with H_2_O (50 mL) to obtain **3f** (0.378 g, 66%) as a white solid, mp 135–138 °C; ^1^H-NMR (DMSO-*d*_6_) δ 3.61 (s, 3H, NCH_3_), 7.08 (ddd, *J =* 8.2, 6.9, 1.3 Hz, 1H, 5-H), 7.38–7.41 (m, 3H, 2’/4’/6’-H), 7.48–7.51 (m, 3H, 4/3’/5’-H), 7.80 (dd, *J =* 7.9, 1.6 Hz, 1H, 6-H), 8.68 (dd, *J =* 8.5, 1.0 Hz, 1H, 3-H), 10.54 (s, 1H, NH), 13.33 (br s, 1H, CO_2_H); ^13^C-NMR (DMSO-*d*_6_) δ 43.23 (NCH_3_), 118.36 (C-1), 122.75, 122.98 (C-3/5), 127.12 (C-2’/6’), 128.45 (C-4’), 130.32 (C-3’/5’), 130.40 (C-6), 132.43 (C-4), 142.13, 143.53 (C-2/1’), 169.33 (CO_2_H), 179.74 (NHCS); Anal. calcd. for C_15_H_14_N_2_O_2_S: C, 62.9; H, 4.9; N, 9.8. Found: C, 62.7; H, 5.1; N, 9.7.

### 2-(3-Benzyl-3-methylthioureido)benzoic acid *(**3g**)*

According to the preparation of **3f**, compound **3g** (0.365 g, 61%) was obtained from **1g** as a white solid, mp 117–119 °C; ^1^H-NMR (DMSO-*d*_6_) δ 3.24 (s, 3H, NCH_3_), 5.19 (s, 2H, C*H*_2_Ph), 7.17 (ddd, *J =* 8.2, 7.3, 1.0 Hz, 1H, 5-H), 7.25–7.29 (m, 5H, 2’/3’/4’/5’/6’-H), 7.54 (ddd, *J =* 8.5, 7.3, 1.3 Hz, 1H, 4-H), 7.92 (dd, *J =* 7.9, 1.3 Hz, 1H, 6-H), 8.44 (dd, *J =* 8.5, 1.0 Hz, 1H, 3-H), 10.75 (s, 1H, NH), 13.46 (br s, 1H, CO_2_H); ^13^C-NMR (DMSO-*d*_6_) δ 37.78 (NCH_3_), 55.92 (*C*H_2_Ph), 120.41 (C-1), 123.34 (C-5), 124.50 (C-3), 127.26 (C-2’/6’), 127.32 (C-4’), 128.66 (C-3’/5’), 130.54 (C-6), 132.42 (C-4), 137.11 (C-2), 142.55 (C-1’), 169.42 (CO_2_H), 180.53 (NHCS); Anal. calcd. for C_16_H_16_N_2_O_2_S: C, 64.0; H, 5.4; N, 9.3. Found: C, 63.8; H, 5.5; N, 9.5.

### 2-[3-Methyl-3-(2-phenylethyl)thioureido]benzoic acid *(**3h**)*

According to the preparation of **3f**, compound **3h** (0.509 g, 81%) was obtained from **1h** as a light yellow solid, mp 130–133 °C; ^1^H-NMR (DMSO-*d*_6_) δ 2.97 (t, *J =* 7.9 Hz, 2H, CH_2_C*H*_2_Ph), 3.25 (s, 3H, NCH_3_), 4.02 (t, *J =* 7.6 Hz, 2H, C*H*_2_CH_2_Ph), 7.12–7.16 (m, 1H, 5-H), 7.19–7.23 (m, 1H, 4-H’), 7.28–7.31 (m, 4H, 2’/3’/5’/6’-H), 7.53 (ddd, *J =* 8.5, 7.3, 1.6 Hz, 1H, 4-H), 7.92 (dd, *J =* 7.9, 1.6 Hz, 1H, 6-H), 8.46 (d, *J =* 7.9 Hz, 1H, 3-H), 10.71 (s, 1H, NH), 13.52 (br s, 1H, CO_2_H); ^13^C-NMR (DMSO-*d*_6_) δ 32.69 (CH_2_*C*H_2_Ph), 54.78 (*C*H_2_CH_2_Ph), 119.45 (C-1), 122.91 (C-5), 123.95 (C-3), 126.42 (C-4’), 128.54, 128.91 (C-2’/3’/5’/6’), 130.52 (C-6), 132.45 (C-4), 138.83 (C-2), 142.69 (C-1’), 169.69 (CO_2_H), 179.32 (NHCS); Anal. calcd. for C_17_H_18_N_2_O_2_S: C, 64.9; H, 5.8; N, 8.9. Found: C, 64.6; H, 6.1; N, 8.7.

### 2-[(Methylthio)thiocarbonylamino]benzoic acid *(**4i**)*

Triethylamine (1.70 g, 16.8 mmol) was added dropwise to an ice-cooled solution of anthranilic acid (0.960 g, 7.0 mmol) and carbon disulfide (1.07 g, 14.0 mmol) in 1,4-dioxane (30 mL). The cooled mixture was stirred for 5.5 h, followed by a dropwise addition of methyl iodide (1.09 g, 7.7 mmol) in 1,4-dioxane (20 mL). After stirring for further 1.5 h in the ice-bath, the reaction mixture was allowed to warm to r.t. and stirred for 21 h under light protection. The solvent was removed under reduced pressure, and the crude material was partionated between EtOAc (100 mL) and HCl (0.2 M, 100 mL). The aqueous phase was further extracted with EtOAc (2 × 200 mL). The combined organic layers were dried (Na_2_SO_4_), filtered and evaporated to dryness. Recrystallisation from PhMe gave **4i** (1.22 g, 77%) as light yellow needles, mp 148–150 °C (PhMe); ^1^H-NMR (DMSO-*d*_6_) δ 2.59 (s, 3H, SCH_3_), 7.36 (td, *J =* 7.9, 1.3 Hz, 1H, 5-H), 7.61 (ddd, *J =* 8.2, 7.0, 1.6 Hz, 1H, 4-H), 7.94 (dd, *J =* 7.9, 1.6 Hz, 1H, 6-H), 8.12 (d, *J =* 7.9 Hz, 1H, 3-H), 11.95 (br s, 1H, NH); ^13^C-NMR (DMSO-*d*_6_) δ 18.11 (SCH_3_), 124.15 (C-1), 125.71 (C-3), 126.37 (C-5), 131.06 (C-6), 132.95 (C-4), 139.93 (C-2), 167.96 (CO_2_H), 198.06 (NHCS); Anal. calcd. for C_9_H_9_NO_2_S_2_: C, 47.6; H, 4.0; N, 6.2. Found: C, 47.3; H, 4.3; N, 6.2.

### 2-[(Ethylthio)thiocarbonylamino]benzoic acid *(**4j**)*

According to the preparation of **4i**, compound **4j** (1.42 g, 84%) was obtained from ethyl iodide as light yellow needles, mp 132–135 °C (PhMe); ^1^H-NMR (DMSO-*d*_6_) δ 1.26 (t, *J =* 7.3 Hz, 3H, CH_2_C*H*_3_), 3.20 (q, *J =* 7.3 Hz, 2H, C*H*_2_CH_3_), 7.36 (td, *J =* 7.6, 1.3 Hz, 1H, 5-H), 7.61 (ddd, *J =* 8.2, 6.9, 1.6 Hz, 1H, 4-H), 7.93 (dd, *J =* 7.9, 1.6 Hz, 1H, 6-H), 8.11 (d, *J =* 8.2 Hz, 1H, 3-H), 11.97 (br s, 1H, NH); ^13^C-NMR (DMSO-*d*_6_) δ 14.06 (CH_2_*C*H_3_), 29.08 (*C*H_2_CH_3_), 124.31 (C-1), 125.73 (C-3), 126.35 (C-5), 131.04 (C-6), 132.90 (C-4), 139.88 (C-2), 167.93 (CO_2_H), 197.11 (NHCS); Anal. calcd. for C_10_H_11_NO_2_S_2_: C, 49.8; H, 4.6; N, 5.8. Found: C, 49.7; H, 4.7; N, 5.8.

### 2-[(Benzylthio)thiocarbonylamino]benzoic acid *(**4k**)*

According to the preparation of **4i**, compound **4k** (1.28 g, 60%) was obtained from benzyl bromide as a yellow solid, mp 142–144 °C (EtOAc); ^1^H-NMR (DMSO-*d*_6_) δ 4.54 (s, 2H, C*H*_2_Ph), 7.25 (td, *J =* 6.6, 1.6 Hz, 1H, H-4’), 7.30–7.34 (m, 2H, 2’/6’-H), 7.35–7.40 (m, 3H, 5/3’/5’-H), 7.62 (ddd, *J =* 8.3, 7.1, 1.6 Hz, 1H, 4-H), 7.94 (dd, *J =* 7.9, 1.6 Hz, 1H, 6-H), 8.03 (d, *J =* 8.5 Hz, 1H, 3-H), 11.98 (br s, 1H, NH); ^13^C-NMR (DMSO-*d*_6_) δ 39.79 (*C*H_2_Ph), 124.71 (C-1), 126.10 (C-3), 126.63 (C-5), 127.43 (C-4’), 128.64 (C-2’/6’), 129.15 (C-3’/5’), 131.07 (C-6), 132.95 (C-4), 136.64 (C-1’), 139.75 (C-2), 167.78 (CO_2_H), 196.67 (NHCS); Anal. calcd. for C_15_H_13_NO_2_S_2_: C, 59.4; H, 4.3; N, 4.6. Found: C, 59.6; H, 4.3; N, 4.65.

### 2-{[(2-Phenylethyl)thio]thiocarbonylamino}benzoic acid *(**4l**)*

According to the preparation of **4i**, compound **4l** (0.527 g, 24%) was obtained from 2-phenylethyl bromide as a yellow solid, mp 125–128 °C (CHCl_3_); ^1^H-NMR (DMSO-*d*_6_) δ 2.94 (t, *J =* 8.2 Hz, 2H, C*H*_2_CH_2_Ph), 3.47 (t, *J =* 7.9 Hz, 2H, CH_2_C*H*_2_Ph), 7.19–7.22 (m, 1H, 4’-H), 7.26–7.32 (m, 4H, 2’/3’/5’/6’-H), 7.37 (td, *J =* 7.9, 1.3 Hz, 1H, 5-H), 7.61 (td, *J =* 7.6, 1.6 Hz, 1H, 4-H), 7.94 (dd, *J =* 7.9, 1.6 Hz, 1H, 6-H), 8.04 (d, *J =* 7.9 Hz, 1H, 3-H), 11.94 (br s, 1H, NH); ^13^C-NMR (DMSO-*d*_6_) δ 34.67 (*C*H_2_CH_2_Ph), 36.00 (CH_2_*C*H_2_Ph), 124.64 (C-1), 126.04 (C-3), 126.49 (C-5/4’), 128.54 (C-2’/6’), 128.67 (C-3’/5’), 131.04 (C-6), 133.83 (C-4), 139.81 (C-1’), 140.13 (C-2), 167.84 (CO_2_H), 196.98 (NHCS); Anal. calcd. for C_16_H_15_NO_2_S_2_: C, 60.5; H, 4.8; N, 4.4. Found: C, 60.2; H, 4.8; N, 5.0.

### 2-(Methylthio)-4*H*-3,1-benzothiazin-4-one *(**5i**)*

*Method 1:* 2-[(Methylthio)thiocarbonylamino]benzoic acid (**4i**, 0.909 g, 4.0 mmol) was heated to reflux in Ac_2_O (10 mL) for 30 min. The solvent was removed under reduced pressure, and the crude material was recrystallised from MeOH to obtain **5i** (0.782 g, 93%) as colourless needles, mp 54–56 °C (MeOH); ^1^H-NMR (DMSO-*d*_6_) δ 2.72 (s, 3H, SCH_3_), 7.58 (ddd, *J =* 7.9, 7.3, 1.3 Hz, 1H, 6-H), 7.72 (dd, *J =* 8.0, 1.2 Hz, 1H, 8-H), 7.92 (ddd, *J =* 8.5, 7.3, 1.9 Hz, 1H, 7-H), 8.06 (dd, *J =* 8.2, 1.9 Hz, 1H, 5-H); ^13^C-NMR (DMSO-*d*_6_) δ 13.92 (SCH_3_), 118.65 (C-4a), 124.68 (C-5), 128.33 (C-6), 129.86 (C-8), 136.84 (C-7), 147.50 (C-8a), 163.47 (C-2), 182.33 (C-4); Anal. calcd. for C_9_H_7_NOS_2_: C, 51.65; H, 3.4; N, 6.7. Found: C, 51.7; H, 3.4; N, 6.7.

*Method 2:* 2-[(Methylthio)thiocarbonylamino]benzoic acid (**4i**, 0.682 g, 3.0 mmol) and TFAA (7.0 mL) were kept at r.t. for 12 h. After removal of the solvent, the resulting crude material was purified by column chromatography on silica using petroleum ether/EtOAc (8+1) as eluent to give **5i** (0.571 g, 91%) as yellowish needles.

### 2-(Ethylthio)-4*H*-3,1-benzothiazin-4-one *(**5j**)*

According to the preparation of **5i** (Method 1), **5j** (0.792 g, 89%) was obtained from **4j** as yellow needles, mp 53–54 °C (EtOH); ^1^H-NMR (DMSO-*d*_6_) δ 1.38 (t, *J =* 7.3 Hz, 3H, SCH_2_C*H*_3_), 3.35 (q, *J =* 7.3 Hz, 2H, SC*H*_2_CH_3_), 7.58 (td, *J =* 7.6, 1.3 Hz, 1H, 6-H), 7.71 (dd, *J =* 8.1, 1.0 Hz, 1H, 8-H), 7.92 (ddd, *J =* 8.1, 6.8, 1.9 Hz, 1H, 7-H), 8.06 (dd, *J =* 7.9, 1.6 Hz, 1H, 5-H); ^13^C-NMR (DMSO-*d*_6_) δ 14.47 (SCH_2_*C*H_3_), 25.83 (S*C*H_2_CH_3_), 118.77 (C-4a), 124.66 (C-5), 128.36 (C-6), 129.89 (C-8), 136.82 (C-7), 147.52 (C-8a), 162.62 (C-2), 182.44 (C-4); Anal. calcd. for C_10_H_9_NOS_2_: C, 53.8; H, 4.1; N, 6.3. Found: C, 54.1; H, 4.2; N, 6.2.

### 2-(Benzylthio)-4*H*-3,1-benzothiazin-4-one *(**5k**)*

According to the preparation of **5i** (Method 1), **5k** (1.01 g, 88%) was obtained from **4k** as white needles, mp 69–72 °C (EtOH); ^1^H-NMR (DMSO-*d*_6_) δ 4.64 (s, 2H, C*H*_2_Ph), 7.24–7.27 (m, 1H, 4’-H), 7.31–7.33 (m, 2H, 3’/5’-H), 7.49–7.50 (m, 2H, 2’/6’-H), 7.59 (ddd, *J =* 7.7, 7.6, 1.3 Hz, 1H, 6-H), 7.80 (dd, *J =* 8.5, 1.3 Hz, 1H, 8-H), 7.94 (ddd, *J =* 8.2, 7.3, 1.6 Hz, 1H, 7-H), 8.06 (dd, *J =* 7.9, 1.3 Hz, 1H, 5-H); ^13^C-NMR (DMSO-*d*_6_) δ 34.88 (*C*H_2_Ph), 118.73 (C-4a), 124.74 (C-5), 127.63 (C-4’), 128.50 (C-6), 128.66 (C-2’/6’), 129.41 (C-3’/5’), 129.88 (C-8), 136.83 (C-7), 136.90 (C-1’), 147.34 (C-8a), 162.03 (C-2), 182.25 (C-4); Anal. calcd. for C_15_H_11_NOS_2_: C, 63.1; H, 3.9; N, 4.9. Found: C, 63.2; H, 3.95; N, 4.9.

### 2-[(2-Phenylethyl)thio]-4*H*-3,1-benzothiazin-4-one *(**5l**)*

According to the preparation of **5i** (Method 1), **5l** (0.671 g, 56%) was obtained from **4l** as pink blocks, mp 63–65 °C (twice from cyclohexane); ^1^H-NMR (CDCl_3_) δ 3.08 (t, *J =* 7.6 Hz, 2H, C*H*_2_CH_2_Ph), 3.55 (t, *J =* 7.9 Hz, 2H, CH_2_C*H*_2_Ph), 7.23–7.34 (m, 5H, 2’/3’/4’/5’/6’-H), 7.44 (td, *J =* 7.4, 1.3 Hz, 1H, 6-H), 7.68 (dd, *J =* 8.7, 1.6 Hz, 1H, 8-H), 7.76 (ddd, *J =* 8.4, 7.0, 1.6 Hz, 1H, 7-H), 8.15 (dd, *J =* 7.9, 1.6 Hz, 1H, 5-H); ^13^C-NMR (CDCl_3_) δ 32.71 (*C*H_2_CH_2_Ph), 35.66 (CH_2_*C*H_2_Ph), 119.46 (C-4a), 125.04 (C-5), 126.73 (C-4’), 127.56 (C-6), 128.62 (C-2’/6’), 128.64 (C-3’/5’), 129.81 (C-8), 135.78 (C-7), 139.69 (C-1’), 148.06 (C-8a), 163.21 (C-2), 183.33 (C-4); Anal. calcd. for C_16_H_13_NOS_2_: C, 64.2; H, 4.4; N, 4.7. Found: C, 64.3; H, 4.4; N, 4.7.

### 2-(3,3-Diethylthioureido)-*N*,*N*-diethylbenzamide *(**6a**)*

Diethylamine (0.914 g, 12.5 mmol) was added dropwise to a solution of 2-(methylthio)-4*H*-3,1-benzothiazin-4-one (**5i**, 1.05 g, 5.0 mmol) in acetone (15 mL). After stirring for 1 h, the mixture was heated to reflux for 1 h, and allowed to cool to r.t. The solvent was removed under reduced pressure. Recrystallisation from EtOH gave **6a** (0.998 g, 65%) as colourless prisms, mp 116–117 °C (EtOH); ^1^H-NMR (DMSO-*d*_6_) δ 1.01–1.08 (m, 6H, CH_2_C*H*_3_), 1.14 (t, *J =* 7.1 Hz, 6H, 2 × CH_2_C*H*_3_), 3.20 (q, *J =* 6.9 Hz, 2H, C*H*_2_CH_3_), 3.37 (q, *J =* 6.9 Hz, 2H, C*H*_2_CH_3_), 3.67 (q, *J =* 6.9 Hz, 4H, C*H*_2_CH_3_), 7.20–7.26 (m, 2H, 5/6-H), 7.33–7.38 (m, 1H, 4-H), 7.41 (d, *J =* 7.9 Hz, 1H, 3-H), 8.67 (s, 1H, NH); ^13^C-NMR (DMSO-*d*_6_) δ 12.44, 12.64, 13.91 (3 × CH_2_*C*H_3_), 38.20, 43.09, 44.88 (3 × *C*H_2_CH_3_), 125.37, 125.84 (C-5/6), 128.58 (C-4), 130.00 (C-3), 134.59 (C-1), 138.17 (C-2), 168.26 (CON), 179.34 (NHCS); Anal. calcd. for C_16_H_25_N_3_OS: C, 62.5; H, 8.2; N, 13.7. Found: C, 62.8; H, 8.1; N, 13.8.

### *N*-[2-(Pyrrolidin-1-ylcarbonyl)phenyl]pyrrolidine-1-carbothioamide *(**6c**)*

According to the preparation of **6a**, compound **6c** (1.13 g, 74%) was obtained from **5i** and pyrrolidine as light yellow prisms, mp 160–162 °C (EtOH); ^1^H-NMR (DMSO-*d*_6_) δ 1.71–2.08 (m, 8H, 3/4/3’’/4’’-H), 3.38–3.70 (m, 8H, 2/5/2’’/5’’-H), 7.16 (td, *J =* 7.6, 1.3 Hz, 1H, 4’-H), 7.38 (td, *J =* 7.9, 1.6 Hz, 1H, 5’-H), 7.42 (dd, *J =* 7.7, 1.4 Hz, 1H, 3’-H), 7.88 (d, *J =* 7.9 Hz, 1H, 6’-H), 9.35 (s, 1H, NH); ^13^C-NMR (DMSO-*d*_6_) δ 24.02, 25.91 (C-3/4/3’’/4’’), 46.03, 49.13 (C-2/5/2’’/5’’), 124.03 (C-4’), 126.53 (C-6’), 127.20 (C-3’), 129.26 (C-5’), 130.52 (C-2’), 138.44 (C-1’), 167.39 (CON), 176.74 (NHCS); Anal. calcd. for C_16_H_21_N_3_OS: C, 63.3; H, 7.0; N, 13.85. Found: C, 63.5; H, 7.1; N, 13.8.

### *N*-[2-(Morpholin-4-ylcarbonyl)phenyl]morpholine-4-carbothioamide *(**6e**)*

*Method 1:* According to the preparation of **6a**, compound **6e** (1.51 g, 90%) was obtained from **5i** and morpholine as colourless prisms, mp 170–173 °C (EtOH); ^1^H-NMR (DMSO-*d*_6_) δ 3.48–3.63 (m, 12H, 2/6/2’/3’/5’/6’-H) 3.85 (t, *J =* 4.6 Hz, 4H, 3/5-H), 7.23–7.29 (m, 3H, 3’/4’/6’-H), 7.39 (ddd, *J* = 7.8, 6.9, 1.9 Hz, 1H, 5’-H), 9.31 (s, 1H, NH); ^13^C-NMR (DMSO-*d*_6_) δ 41.70, 47.48, 48.85, 66.06, 66.12, 66.30 (C-2/3/5/6/2’’/3’’/5’’/6’’), 125.75 (C-3’), 127.26 (C-4’), 129.29 (C-5’), 129.35 (C-6’), 133.38 (C-2’), 138.38 (C-1’), 167.06 (CON), 181.93 (NHCS); Anal. calcd. for C_16_H_21_N_3_O_3_S: C, 57.3; H, 6.3; N, 12.5. Found: C, 57.5; H, 6.35; N, 12.35.

*Method 2:* Morpholine (0.392 g, 4.5 mmol) was added dropwise to a solution of 2-(morpholin-4-yl)-4*H*-3,1-benzothiazin-4-one (**2e**, 0.497 g, 2.0 mmol) in acetone (6 mL). After stirring for 1 h, the mixture was heated to reflux for 1 h, and allowed to cool to r.t. The formed precipitate was removed by suction filtration and washed with cold acetone (5 mL) to give **6e** (0.490 g, 73%) as white solid.

### Methyl 2-(morpholin-4-ylcarbonyl)phenyldithiocarbamate *(**7**)*

Morpholine (0.392 g, 4.5 mmol) was added dropwise to a solution of 2-(methylthio)-4*H*-3,1-benzothiazin-4-one (**5i**, 0.418 g, 2.0 mmol) in acetone (6 mL). After stirring for 1 h, the resulting precipitate was removed by suction filtration and washed with cold acetone (5 mL) to obtain **7** (0.299 g, 50%) as a white solid, mp 143–145 °C; ^1^H-NMR (DMSO-*d*_6_) δ 2.55 (s, 3H, SCH_3_), 3.15–3.22, 3.45–3.64 (m, 8H, 2’/3’/5’/6’-H), 7.35–7.49 (m, 4H, 3/4/5/6-H), 11.52 (s, 1H, NH); ^13^C-NMR (DMSO-*d*_6_) δ 18.24 (SCH_3_), 41.90, 47.39 (C-3’’/5’’), 66.09, 66.13 (C-2’’/6’’), 127.50, 127.98 (C-3’/4’), 128.65, 129.77 (C-5’/6’), 133.22 (C-2’), 136.57 (C-1’), 168.40 (CO), 199.94 (NHCS); Anal. calcd. for C_13_H_16_N_2_O_2_S_2_: C, 52.7; H, 5.4; N, 9.45. Found: C, 52.8; H, 5.45; N, 9.5.

### 2-[*N*-Methyl-*N*-(2-phenylethyl)amino]-4*H*-3,1-benzoxazin-4-one *(**8h**)*

In the course of the preparation of **2h** (Method 2) using TFAA, purification of the crude material by column chromatography on silica gave **8h** (0.570 g, 68%) as a white solid, mp 68–70 °C, lit. [8] 68.5–69 °C; ^1^H-NMR (CDCl_3_) δ 2.94 (t, *J =* 7.3 Hz, 2H, CH_2_C*H*_2_Ph), 3.05 (s, 3H, NCH_3_), 3.75 (t, *J =* 7.3 Hz, 2H, C*H*_2_CH_2_Ph), 7.07–7.12 (m, 1H, 6-H), 7.18–7.30 (m, 6H, 8/2’/3’/4’/5’/6’-H), 7.58 (ddd, *J =* 7.8, 7.4, 1.8 Hz, 1H, 7-H), 7.97 (dd, *J =* 7.9, 1.6 Hz, 1H, 5-H); ^13^C-NMR (CDCl_3_) δ 34.01 (CH_2_*C*H_2_Ph), 35.68 (NCH_3_), 51.35 (*C*H_2_CH_2_Ph), 112.06 (C-4a), 123.00, 124.15, 128.61 (C-5/6/8), 126.54 (C-4’), 128.57 (C-2’/6’), 128.82 (C-3’/5’), 136.56 (C-7), 138.42 (C-1’), 150.93 (C-8a), 153.84 (C-2), 159.90 (C-4); Anal. calcd. for C_17_H_16_N_2_O_2_: C, 72.8; H, 5.75; N, 10.0. Found: C, 72.4; H, 6.05; N, 9.7.

### 2-(Methylthio)-4*H*-3,1-benzoxazin-4-one *(**9i**)*

In the course of the preparation of **5i** (Method 2) using TFAA, purification of the crude material by column chromatography on silica yielded **9i** (0.010 g, 2%) as a white solid, mp 103–105 °C, lit. [3] 108–109 °C; ^1^H-NMR (CDCl_3_) δ 2.58 (s, 3H, SCH_3_), 7.40 (ddd, *J =* 8.2, 7.2, 1.3 Hz, 1H, 6-H), 7.45 (d, *J =* 7.9 Hz, 1H, 8-H), 7.74 (ddd, *J =* 7.9, 7.3, 1.6 Hz, 1H, 7-H), 8.11 (dd, *J =* 8.0, 1.6 Hz, 1H, 5-H); ^13^C-NMR (CDCl_3_) δ 14.16 (SCH_3_), 115.59 (C-4a), 125.59, 127.22, 128.77 (C-5/6/8), 136.76 (C-7), 146.89 (C-8a), 158.80 (C-4), 164.00 (C-2).

### HLE inhibition assay

Human leukocyte elastase was assayed spectrophotometrically at 405 nm at 25 °C [49]. Assay buffer was 50 mM sodium phosphate buffer, 500 mM NaCl, pH 7.8. An enzyme stock solution of 50 µg/mL was prepared in 100 mM sodium acetate buffer, pH 5.5 and diluted with assay buffer. Inhibitor stock solutions were prepared in DMSO. A stock solution of the chromogenic substrate MeOSuc-Ala-Ala-Pro-Val-pNA was prepared in DMSO and diluted with assay buffer. The final concentration of HLE was 50 ng/mL, of the chromogenic substrate MeOSuc-Ala-Ala-Pro-Val-pNA was 100 µM, and of DMSO was 5.5%. Into a cuvette containing 870 µL assay buffer, 50 µL of an inhibitor solution and 50 µL of the substrate solution were added and thoroughly mixed. The reaction was initiated by adding 50 µL of the HLE solution and was followed over 10 min. IC_50_ values were calculated from the linear steady-state turnover of the substrate.

### Cathepsin G inhibition assay

Human cathepsin G was assayed spectrophotometrically at 405 nm at 25 °C [7, 8]. Assay buffer was 20 mM Tris HCl buffer, 150 mM NaCl, pH 8.4. Inhibitor stock solutions were prepared in DMSO. An enzyme stock solution of 200 mU/mL was prepared in 50 mM sodium acetate buffer, 150 mM NaCl, pH 5.5. A 50 mM stock solution of the chromogenic substrate Suc-Ala-Ala-Pro-Phe-pNA in DMSO was diluted with assay buffer. The final concentration of cathepsin G was 2.5 mU/mL, of the substrate Suc-Ala-Ala-Pro-Phe-NHNp was 500 µM, and of DMSO was 1.5%. Into a cuvette containing 882.5 µL assay buffer, 5 µL of an inhibitor solution and 100 µL of a substrate solution were added and thoroughly mixed. The reaction was initiated by adding 12.5 µL of the cathepsin G solution and was followed over 10 min. IC_50_ values were calculated from the linear steady-state turnover of the substrate.

### Chymotrypsin inhibition assay

Bovine chymotrypsin was assayed spectrophotometrically at 405 nm at 25 °C. Assay buffer was 20 mM Tris HCl buffer, 150 mM NaCl, pH 8.4. Inhibitor stock solutions were prepared in DMSO. An enzyme stock solution was prepared in 1 mM HCl and diluted with assay buffer. A 40 mM stock solution of the chromogenic substrate Suc-Ala-Ala-Pro-Phe-pNA in DMSO was diluted with assay buffer. The final concentration of chymotrypsin was 12.5 ng/mL, of the substrate Suc-Ala-Ala-Pro-Phe-NHNp was 200 µM, and of DMSO was 6%. Into a cuvette containing 845 µL assay buffer, 55 µL of an inhibitor solution and 50 µL of a substrate solution were added and thoroughly mixed. The reaction was initiated by adding 50 µL of a chymotrypsin solution and was followed over 12.5 min. IC_50_ values were calculated from the linear steady-state turnover of the substrate.

### Trypsin inhibition assay

Trypsin from bovine pancreas was assayed spectrophotometrically at 405 nm at 25 °C. Assay buffer was 20 mM Tris HCl buffer, 150 mM NaCl, pH 8.4. An enzyme stock solution of 10 µg/mL was prepared in 1 mM HCl and diluted with assay buffer. Inhibitor stock solutions were prepared in DMSO. A 40 mM stock solution of the chromogenic substrate Suc-Ala-Ala-Pro-Arg-pNA in DMSO was diluted with assay buffer. The final concentration of trypsin was 12.5 ng/mL, of the substrate Suc-Ala-Ala-Pro-Arg-pNA was 200 µM, and of DMSO was 6%. Into a cuvette containing 845 µL assay buffer, 55 µL of an inhibitor solution and 50 µL of a substrate solution were added and thoroughly mixed. The reaction was initiated by adding 50 µL of the trypsin solution and was followed over 12.5 min. IC_50_ values were calculated from the linear steady-state turnover of the substrate.

### Cathepsin L inhibition assay

Human cathepsin L was assayed spectrophotometrically at 405 nm at 37 °C [50]. Assay buffer was 100 mM sodium phosphate buffer, pH 6.0, 100 mM NaCl, 5 mM EDTA, 0.01% Brij 35. An enzyme stock solution of 50 µg/mL in 20 mM sodium acetate buffer, pH 5.0, 100 mM NaCl, 10 mM trehalose, 1 mM EDTA, 50% glycerol was diluted 1:100 with assay buffer containing 5 mM DTT and incubated for 30 min at 37 °C. This enzyme solution was diluted 1:5 with assay buffer containing 5 mM DTT. Inhibitor stock solutions were prepared in DMSO. A 10 mM stock solution of the chromogenic substrate Z-Phe-Arg-pNA was prepared with DMSO. The final concentration of cathepsin L was 4 ng/mL, of the substrate Z-Phe-Arg-pNA was 100 µM, and of DMSO was 5%. Into a cuvette containing 910 µL assay buffer, 40 µL of an inhibitor solution and 10 µL of a substrate solution were added and thoroughly mixed. The reaction was initiated by adding 40 µL of the cathepsin L solution and was followed over 10 min. IC_50_ values were calculated from the linear steady-state turnover of the substrate.

### ACE inhibition assay

Human ACE was assayed spectrophotometrically at 352 nm at 37 °C [51]. Assay buffer was 50 mM Tris HCl buffer, 300 mM NaCl, pH 7.5. An enzyme stock solution of 434 µg/mL in 12.5 mM HCl, pH 7.5, 75 mM NaCl, 500 nM ZnCl_2_, 40% glycerol was diluted 1:100 with assay buffer. After incubation for 10 min at 37 °C, the enzyme solution was stored at 0 °C and used within 90 min. Inhibitor stock solutions were prepared in DMSO. A 300 mM stock solution of the chromogenic substrate FA-Phe-Gly-Gly was prepared in DMSO. The final concentration of ACE was 86.8 ng/mL, of the substrate FA-Phe-Gly-Gly was 3 mM, and of DMSO was 3%. Into a cuvette containing 950 µL assay buffer, 20 µL of an inhibitor solution and 10 µL of a substrate solution were added and thoroughly mixed. The reaction was initiated by adding 20 µL of the ACE solution and was followed over 20 min. IC_50_ values were calculated from the linear steady-state turnover of the substrate.

### AChE inhibition assay

Acetylcholinesterase inhibition was assayed spectrophotometrically at 412 nm at 25 °C [52,53,54]. Assay buffer was 100 mM sodium phosphate, 100 mM NaCl, pH 7.3. The enzyme stock solution (~100 U/mL) in assay buffer was kept at 0 °C. Appropriate dilutions were prepared immediately before starting the measurement. ATCh (10 mM) and DTNB (7 mM) were dissolved in assay buffer and kept at 0 °C. Stock solutions of the test compounds were prepared in acetonitrile. The final concentration of AChE was ~30 mU/mL, of ATCh was 500 µM, of DTNB was 350 µM, and of acetonitrile was 6%. Into a cuvette containing 830 µL assay buffer, 50 µL of the DTNB solution, 50 µL acetonitrile, 10 µL of a solution of the test compound, and 10 µL of an enzyme solution (~3 U/mL) were added and thoroughly mixed. After incubation for 15 min at 25 °C, the reaction was initiated by adding 50 µL of the ATCh solution and was followed over 5 min. IC_50_ values were calculated from the linear steady-state turnover of the substrate.

### CEase inhibition assay

Cholesterol esterase inhibition was assayed spectrophotometrically at 405 nm at 25 °C [55, 56]. Assay buffer was 100 mM sodium phosphate, 100 mM NaCl, pH 7.0. A stock solution of CEase was prepared in 100 mM sodium phosphate buffer, pH 7.0 and kept at 0 °C. A 1:122 dilution was done immediately before starting the measurement. TC (12 mM) was dissolved in assay buffer and kept at 25 °C. Stock solutions of all test compounds and of pNPB (20 mM) were prepared in acetonitrile. The final concentration of CEase was 10 ng/mL, of the substrate pNPB was 200 µM, of TC was 6 mM, and of acetonitrile was 6%. Into a cuvette containing 430 µL assay buffer, 500 µL of the TC solution, 40 µL acetonitrile, 10 µL of the pNPB solution, and 10 µL of a solution of the test compound were added and thoroughly mixed. After incubation for 5 min at 25 °C, the reaction was initiated by adding 10 µL of the enzyme solution (1 µg/mL). IC_50_ values were calculated from the linear steady-state turnover of the substrate.

## Electronic Supplementary Information (ESI) available

Enzyme kinetic data, crystallographic details, and selected two-dimensional NMR spectra (compounds **2c**, **2g**, **4i**, **5i**, **6a**, **6c**, and **6e**).
