# Supplementary material for: 2-Amino- and 2-Alkylthio-4H-3,1-benzothiazin-4-ones: Synthesis, Interconversion and Enzyme Inhibitory Activities
Source: Molecules. 2009 Jan 14;14(1):378–402. doi: 10.3390/molecules14010378 (PMC6253953; doi:10.3390/molecules14010378)

Molecules **2009**, *14*, 378-402; doi:10.3390/molecules14010378

OPEN ACCESS

molecules

ISSN 1420-3049

www.mdpi.com/journal/molecules

Supporting Information

2-Amino- and 2-Alkylthio-4*H*-3,1-benzothiazin-4-ones: Synthesis, Interconversion and Enzyme Inhibitory Activities

Hans-Georg Häcker [1], Florian Grundmann [1], Friederike Lohr [1], Philipp A. Ottersbach [1], Jing Zhou [1], Gregor Schnakenburg [2] and Michael Gütschow [1,*]

[1] Pharmaceutical Institute, Pharmaceutical Chemistry I, University of Bonn, An der Immenburg 4, D-53121 Bonn, Germany

[2] Institute of Inorganic Chemistry, University of Bonn, Gerhard-Domagk-Straße 1, D-53121 Bonn, Germany

* Author to whom correspondence should be addressed. E-mail: guetschow@uni-bonn.de, Tel: +49 228 732317, Fax: +49 228 732567.

Received: / Accepted: / Published:

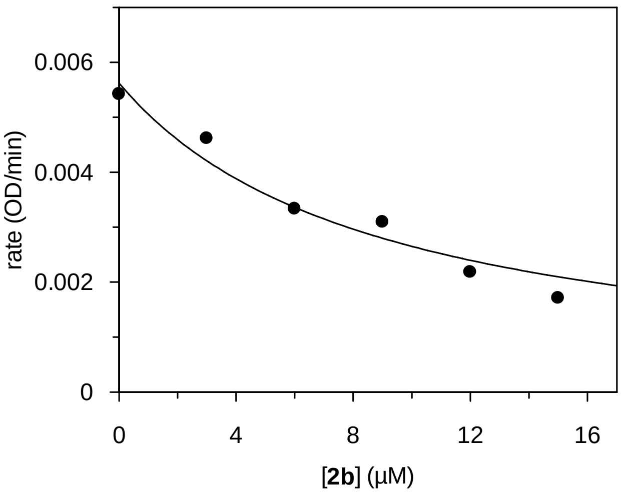

Fig. S1 Plot of the steady-state rates *versus* inhibitor concentration for the inhibition of human cathepsin L by compound **2b**.

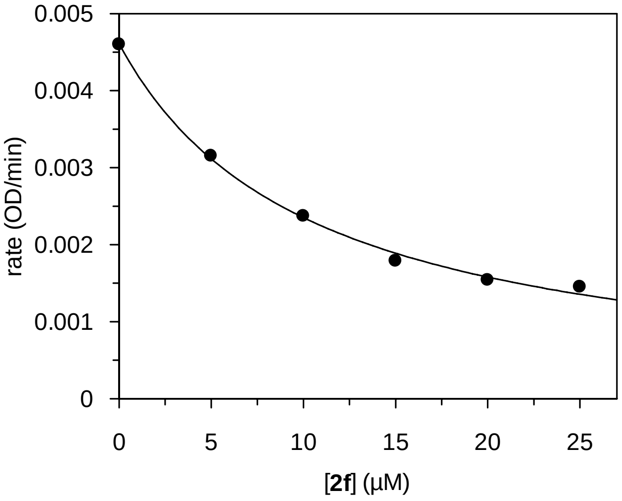

Fig. S2 Plot of the steady-state rates *versus* inhibitor concentration for the inhibition of bovine chymotrypsin by compound **2f**.

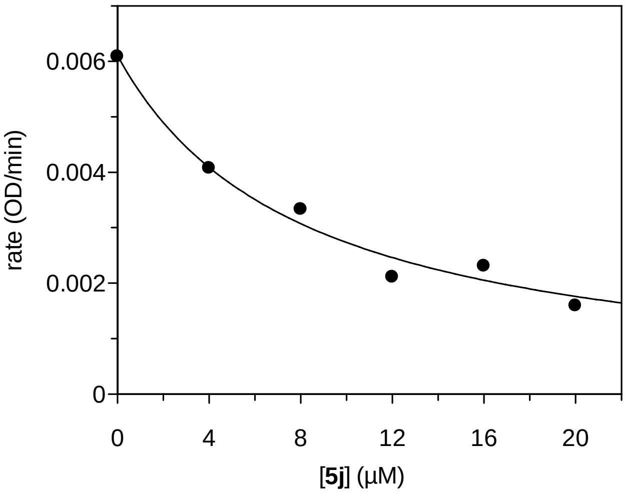

Fig. S3 Plot of the steady-state rates *versus* inhibitor concentration for the inhibition of human leukocyte elastase by compound **5j**.

Table S1 Crystal data and structure refinement for 2-(*N*-benzyl-*N*-methylamino)-4*H*-3,1-benzothiazin-4-one **2g**.

Device Type	Nonius KappaCCD	
Empirical formula	$C_{16}H_{14}N_2OS$	
Formula weight	282.35	
Temperature	123(2) K	
Wavelength	0.71073 Å	
Crystal system, space group	Triclinic, P-1	
Unit cell dimensions	a = 5.2288(3) Å	α = 95.276(3)°
	b = 9.5012(5) Å	β = 92.293(3)°
	c = 13.6203(9) Å	γ = 94.088(4)°
Volume	671.33(7) $Å^3$	
Z, Calculated density	2, 1.397 Mg/m^3	
Absorption coefficient	0.237 mm^{-1}	
F(000)	296	
Crystal size	0.60 × 0.24 × 0.20 mm	
Theta range for data collection	2.51 to 27.88°	
Limiting indices	-6<=h<=6, -12<=k<=11, -16<=l<=17	
Reflections collected / unique	7138 / 3040 [R(int) = 0.0569]	
Completeness to theta = 27.88	95.0%	
Absorption correction	Semi-empirical from equivalents	
Max. and min. transmission	0.97987 and 0.87043	
Refinement method	Full-matrix least-squares on F^2	
Data / restraints / parameters	3040 / 0 / 182	
Goodness-of-fit on F^2	1.067	
Final R indices [I>2sigma(I)]	R1 = 0.0398, wR2 = 0.1018	
R indices (all data)	R1 = 0.0507, wR2 = 0.1074	
Largest diff. peak and hole	0.339 and -0.485 e.$Å^{-3}$	

Table S2 Atomic coordinates (× 10^4) and equivalent isotropic displacement parameters ($Å^2$ × 10^3) for **2g**.

U(eq) is defined as one third of the trace of the orthogonalized U^{ij} tensor.

	x	y	z	U(eq)
C(1)	1545(3)	4282(1)	3011(1)	16(1)
C(2)	-25(3)	2655(2)	4513(1)	21(1)
C(3)	-1920(3)	2095(1)	3731(1)	19(1)
C(4)	-3751(3)	1041(2)	3965(1)	25(1)
C(5)	-5707(3)	506(2)	3299(1)	25(1)
C(6)	-5837(3)	1020(2)	2373(1)	23(1)
C(7)	-4038(3)	2038(1)	2118(1)	21(1)
C(8)	-2008(3)	2613(1)	2790(1)	17(1)
C(9)	3136(3)	5443(2)	1609(1)	19(1)
C(10)	1488(3)	6600(1)	1314(1)	18(1)
C(11)	1133(3)	6796(2)	320(1)	24(1)
C(12)	-385(3)	7830(2)	21(1)	28(1)
C(13)	-1594(3)	8680(2)	719(1)	26(1)
C(14)	-1235(3)	8499(2)	1707(1)	24(1)
C(15)	289(3)	7460(1)	2007(1)	20(1)
C(16)	5095(3)	6141(2)	3298(1)	21(1)
N(1)	-329(2)	3633(1)	2452(1)	18(1)
N(2)	3196(2)	5252(1)	2659(1)	19(1)
O(1)	105(2)	2278(1)	5344(1)	32(1)
S(1)	2270(1)	4031(1)	4261(1)	21(1)

Table S3 Bond lengths [Å] and angles [°] for **2g**.

C(1)-N(1)	1.2996(17)	C(9)-C(10)	1.5167(18)
C(1)-N(2)	1.3509(19)	C(9)-H(9B)	0.9900
C(1)-S(1)	1.7708(14)	C(9)-H(9A)	0.9900
C(2)-O(1)	1.2187(18)	C(10)-C(15)	1.3895(19)
C(2)-C(3)	1.4614(19)	C(10)-C(11)	1.390(2)
C(2)-S(1)	1.7774(16)	C(11)-C(12)	1.3856(19)
C(3)-C(4)	1.403(2)	C(11)-H(11A)	0.9500
C(3)-C(8)	1.415(2)	C(12)-C(13)	1.392(2)
C(4)-C(5)	1.376(2)	C(12)-H(12A)	0.9500
C(4)-H(4A)	0.9500	C(13)-C(14)	1.379(2)
C(5)-C(6)	1.393(2)	C(13)-H(13A)	0.9500
C(5)-H(5A)	0.9500	C(14)-C(15)	1.3922(19)
C(6)-C(7)	1.378(2)	C(14)-H(14A)	0.9500
C(6)-H(6A)	0.9500	C(15)-H(15A)	0.9500
C(7)-C(8)	1.4173(19)	C(16)-N(2)	1.4606(17)
C(7)-H(7A)	0.9500	C(16)-H(16C)	0.9800
C(8)-N(1)	1.3837(19)	C(16)-H(16B)	0.9800
C(9)-N(2)	1.4578(18)	C(16)-H(16A)	0.9800
N(1)-C(1)-N(2)	120.91(13)	H(9B)-C(9)-H(9A)	107.6
N(1)-C(1)-S(1)	126.30(12)	C(15)-C(10)-C(11)	118.82(12)
N(2)-C(1)-S(1)	112.79(9)	C(15)-C(10)-C(9)	122.00(13)
O(1)-C(2)-C(3)	125.64(14)	C(11)-C(10)-C(9)	119.17(12)
O(1)-C(2)-S(1)	116.01(11)	C(12)-C(11)-C(10)	120.94(13)
C(3)-C(2)-S(1)	118.33(11)	C(12)-C(11)-H(11A)	119.5
C(4)-C(3)-C(8)	120.51(13)	C(10)-C(11)-H(11A)	119.5
C(4)-C(3)-C(2)	116.80(13)	C(11)-C(12)-C(13)	119.90(14)
C(8)-C(3)-C(2)	122.65(13)	C(11)-C(12)-H(12A)	120.1
C(5)-C(4)-C(3)	121.21(15)	C(13)-C(12)-H(12A)	120.1
C(5)-C(4)-H(4A)	119.4	C(14)-C(13)-C(12)	119.50(13)
C(3)-C(4)-H(4A)	119.4	C(14)-C(13)-H(13A)	120.3
C(4)-C(5)-C(6)	118.86(14)	C(12)-C(13)-H(13A)	120.3
C(4)-C(5)-H(5A)	120.6	C(13)-C(14)-C(15)	120.57(13)
C(6)-C(5)-H(5A)	120.6	C(13)-C(14)-H(14A)	119.7
C(7)-C(6)-C(5)	121.13(13)	C(15)-C(14)-H(14A)	119.7
C(7)-C(6)-H(6A)	119.4	C(10)-C(15)-C(14)	120.26(14)
C(5)-C(6)-H(6A)	119.4	C(10)-C(15)-H(15A)	119.9
C(6)-C(7)-C(8)	121.30(14)	C(14)-C(15)-H(15A)	119.9
C(6)-C(7)-H(7A)	119.4	N(2)-C(16)-H(16C)	109.5
C(8)-C(7)-H(7A)	119.4	N(2)-C(16)-H(16B)	109.5
N(1)-C(8)-C(3)	126.97(12)	H(16C)-C(16)-H(16B)	109.5
N(1)-C(8)-C(7)	116.06(13)	N(2)-C(16)-H(16A)	109.5
C(3)-C(8)-C(7)	116.97(14)	H(16C)-C(16)-H(16A)	109.5
N(2)-C(9)-C(10)	114.45(11)	H(16B)-C(16)-H(16A)	109.5

Table S3 (continued).

N(2)-C(9)-H(9B)	108.6	C(1)-N(1)-C(8)	121.96(12)
C(10)-C(9)-H(9B)	108.6	C(1)-N(2)-C(9)	120.39(11)
N(2)-C(9)-H(9A)	108.6	C(1)-N(2)-C(16)	122.25(12)
C(10)-C(9)-H(9A)	108.6	C(9)-N(2)-C(16)	117.36(12)
		C(1)-S(1)-C(2)	103.69(7)

Table S4 Anisotropic displacement parameters (Å^2 × 10^3) for **2g**.

The anisotropic displacement factor exponent takes the form: $-2 \pi^2 [h^2 a^{*2} U^{11} + ... + 2 h k a^* b^* U^{12}]$

	U11	U22	U33	U23	U13	U12
C(1)	18(1)	16(1)	15(1)	1(1)	-1(1)	5(1)
C(2)	23(1)	23(1)	18(1)	3(1)	2(1)	1(1)
C(3)	19(1)	20(1)	19(1)	1(1)	2(1)	2(1)
C(4)	27(1)	25(1)	22(1)	4(1)	4(1)	0(1)
C(5)	22(1)	21(1)	32(1)	3(1)	3(1)	-2(1)
C(6)	20(1)	21(1)	28(1)	0(1)	-4(1)	2(1)
C(7)	21(1)	20(1)	22(1)	2(1)	-4(1)	5(1)
C(8)	18(1)	17(1)	18(1)	0(1)	0(1)	5(1)
C(9)	22(1)	23(1)	14(1)	3(1)	3(1)	2(1)
C(10)	19(1)	17(1)	18(1)	2(1)	-1(1)	-3(1)
C(11)	34(1)	21(1)	18(1)	1(1)	1(1)	1(1)
C(12)	38(1)	25(1)	20(1)	6(1)	-6(1)	-1(1)
C(13)	26(1)	20(1)	33(1)	8(1)	-5(1)	0(1)
C(14)	24(1)	21(1)	28(1)	2(1)	3(1)	1(1)
C(15)	21(1)	21(1)	17(1)	3(1)	0(1)	-2(1)
C(16)	20(1)	24(1)	20(1)	2(1)	-3(1)	-3(1)
N(1)	19(1)	19(1)	16(1)	3(1)	-2(1)	2(1)
N(2)	19(1)	22(1)	15(1)	3(1)	-2(1)	-1(1)
O(1)	40(1)	36(1)	18(1)	7(1)	-2(1)	-9(1)
S(1)	23(1)	24(1)	15(1)	3(1)	-2(1)	-2(1)

Table S5 Hydrogen coordinates (× 10^4) and isotropic displacement parameters (Å^2 × 10^3) for **2g**.

	x	y	z	U(eq)
H(4A)	-3639	689	4595	29
H(5A)	-6948	-201	3466	30
H(6A)	-7188	663	1911	28
H(7A)	-4161	2362	1480	25
H(9B)	2490	4537	1234	23
H(9A)	4913	5667	1415	23
H(11A)	1942	6214	-160	29
H(12A)	-600	7957	-661	33
H(13A)	-2658	9381	516	32
H(14A)	-2034	9089	2186	29
H(15A)	509	7338	2689	24
H(16C)	5691	6963	2961	32
H(16B)	6553	5592	3449	32
H(16A)	4313	6465	3913	32

Table S6 Torsion angles [°] for **2g**.

O(1)-C(2)-C(3)-C(4)	0.4(2)
S(1)-C(2)-C(3)-C(4)	-178.09(10)
O(1)-C(2)-C(3)-C(8)	178.00(13)
S(1)-C(2)-C(3)-C(8)	-0.51(18)
C(8)-C(3)-C(4)-C(5)	-1.5(2)
C(2)-C(3)-C(4)-C(5)	176.12(13)
C(3)-C(4)-C(5)-C(6)	0.6(2)
C(4)-C(5)-C(6)-C(7)	0.5(2)
C(5)-C(6)-C(7)-C(8)	-0.8(2)
C(4)-C(3)-C(8)-N(1)	-179.27(11)
C(2)-C(3)-C(8)-N(1)	3.2(2)
C(4)-C(3)-C(8)-C(7)	1.23(19)
C(2)-C(3)-C(8)-C(7)	-176.26(11)
C(6)-C(7)-C(8)-N(1)	-179.68(11)
C(6)-C(7)-C(8)-C(3)	-0.12(19)
N(2)-C(9)-C(10)-C(15)	4.1(2)
N(2)-C(9)-C(10)-C(11)	-174.94(12)
C(15)-C(10)-C(11)-C(12)	0.1(2)
C(9)-C(10)-C(11)-C(12)	179.18(13)
C(10)-C(11)-C(12)-C(13)	-0.5(2)
C(11)-C(12)-C(13)-C(14)	1.0(2)
C(12)-C(13)-C(14)-C(15)	-1.1(2)
C(11)-C(10)-C(15)-C(14)	-0.1(2)
C(9)-C(10)-C(15)-C(14)	-179.20(13)
C(13)-C(14)-C(15)-C(10)	0.6(2)
N(2)-C(1)-N(1)-C(8)	178.52(11)
S(1)-C(1)-N(1)-C(8)	-1.05(18)
C(3)-C(8)-N(1)-C(1)	-2.5(2)
C(7)-C(8)-N(1)-C(1)	177.06(11)
N(1)-C(1)-N(2)-C(9)	-8.87(18)
S(1)-C(1)-N(2)-C(9)	170.76(9)
N(1)-C(1)-N(2)-C(16)	171.28(12)
S(1)-C(1)-N(2)-C(16)	-9.10(15)
C(10)-C(9)-N(2)-C(1)	93.62(15)
C(10)-C(9)-N(2)-C(16)	-86.52(14)
N(1)-C(1)-S(1)-C(2)	2.94(13)
N(2)-C(1)-S(1)-C(2)	-176.66(9)
O(1)-C(2)-S(1)-C(1)	179.37(11)
C(3)-C(2)-S(1)-C(1)	-1.99(12)

Table S7 Crystal data and structure refinement for 2-(benzylthio)-4*H*-3,1-benzothiazin-4-one **5k**.

Device Type	Nonius KappaCCD	
Empirical formula	$C_{15}H_{11}NOS_2$	
Formula weight	285.37	
Temperature	123(2) K	
Wavelength	0.71073 Å	
Crystal system, space group	Monoclinic, P 21/a	
Unit cell dimensions	a = 15.7886(14) Å	α = 90°
	b = 5.5513(3) Å	β = 110.554(3)°
	c = 15.8381(14) Å	γ = 90°
Volume	1299.80(18) $Å^3$	
Z, Calculated density	4, 1.458 Mg/m^3	
Absorption coefficient	0.399 mm^{-1}	
F(000)	592	
Crystal size	0.44 × 0.12 × 0.10 mm	
Theta range for data collection	2.61 to 27.88°	
Limiting indices	-20<=h<=13, -7<=k<=6, -20<=l<=20	
Reflections collected / unique	7647 / 3013 [R(int) = 0.0395]	
Completeness to theta = 27.88	97.4%	
Absorption correction	Semi-empirical from equivalents	
Max. and min. transmission	0.96481 and 0.92502	
Refinement method	Full-matrix least-squares on F^2	
Data / restraints / parameters	3013 / 0 / 217	
Goodness-of-fit on F^2	0.969	
Final R indices [I>2sigma(I)]	R1 = 0.0355, wR2 = 0.0711	
R indices (all data)	R1 = 0.0614, wR2 = 0.0784	
Largest diff. peak and hole	0.317 and -0.312 e. $Å^{-3}$	

Table S8 Atomic coordinates (× 10^4) and equivalent isotropic displacement parameters ($Å^2$ × 10^3) for **5k**.

U(eq) is defined as one third of the trace of the orthogonalized U^{ij} tensor.

	x	y	z	U(eq)
C(1)	4340(1)	12049(3)	2819(1)	18(1)
C(2)	4570(1)	15934(3)	3981(1)	19(1)
C(3)	5418(1)	16053(3)	3811(1)	18(1)
C(4)	6026(1)	17900(3)	4242(1)	21(1)
C(5)	6856(1)	18064(3)	4147(1)	25(1)
C(6)	7107(1)	16397(3)	3620(1)	25(1)
C(7)	6507(1)	14603(3)	3176(1)	23(1)
C(8)	5660(1)	14395(3)	3259(1)	17(1)
C(9)	4262(2)	8528(3)	1530(1)	23(1)
C(10)	3957(1)	9618(3)	603(1)	19(1)
C(11)	4328(1)	11766(3)	441(1)	24(1)
C(12)	4062(1)	12721(3)	-417(1)	28(1)
C(13)	3429(1)	11547(3)	-1129(1)	28(1)
C(14)	3048(1)	9431(3)	-977(1)	30(1)
C(15)	3308(1)	8473(3)	-116(1)	25(1)
N(1)	5112(1)	12510(2)	2770(1)	19(1)
O(1)	4336(1)	17291(2)	4463(1)	24(1)
S(1)	3790(1)	13566(1)	3461(1)	23(1)
S(2)	3643(1)	9685(1)	2224(1)	22(1)

Table S9 Bond lengths [Å] and angles [°] for **5k**.

C(1)-N(1)	1.275(2)	C(8)-N(1)	1.405(2)
C(1)-S(2)	1.7587(17)	C(9)-C(10)	1.502(2)
C(1)-S(1)	1.7638(17)	C(9)-S(2)	1.8254(19)
C(2)-O(1)	1.2176(19)	C(9)-H(9A)	0.957(16)
C(2)-C(3)	1.458(2)	C(9)-H(9B)	1.006(19)
C(2)-S(1)	1.7917(18)	C(10)-C(15)	1.389(2)
C(3)-C(4)	1.407(2)	C(10)-C(11)	1.392(2)
C(3)-C(8)	1.411(2)	C(11)-C(12)	1.380(3)
C(4)-C(5)	1.373(3)	C(11)-H(11)	0.961(17)
C(4)-H(4)	0.948(17)	C(12)-C(13)	1.379(3)
C(5)-C(6)	1.394(3)	C(12)-H(12)	0.946(17)
C(5)-H(5)	0.899(17)	C(13)-C(14)	1.379(3)
C(6)-C(7)	1.384(3)	C(13)-H(13)	0.946(18)
C(6)-H(6)	0.96(2)	C(14)-C(15)	1.386(3)
C(7)-C(8)	1.394(2)	C(14)-H(14)	0.91(2)
C(7)-H(7)	0.949(18)	C(15)-H(15)	0.947(16)
N(1)-C(1)-S(2)	123.23(13)	C(10)-C(9)-H(9A)	110.7(10)
N(1)-C(1)-S(1)	127.87(13)	S(2)-C(9)-H(9A)	103.2(11)
S(2)-C(1)-S(1)	108.90(10)	C(10)-C(9)-H(9B)	110.6(10)
O(1)-C(2)-C(3)	126.04(16)	S(2)-C(9)-H(9B)	107.1(10)
O(1)-C(2)-S(1)	115.59(14)	H(9A)-C(9)-H(9B)	111.9(15)
C(3)-C(2)-S(1)	118.36(12)	C(15)-C(10)-C(11)	118.63(16)
C(4)-C(3)-C(8)	119.30(17)	C(15)-C(10)-C(9)	120.47(15)
C(4)-C(3)-C(2)	117.22(15)	C(11)-C(10)-C(9)	120.89(16)
C(8)-C(3)-C(2)	123.46(15)	C(12)-C(11)-C(10)	120.55(17)
C(5)-C(4)-C(3)	120.63(17)	C(12)-C(11)-H(11)	121.2(10)
C(5)-C(4)-H(4)	122.7(11)	C(10)-C(11)-H(11)	118.2(10)
C(3)-C(4)-H(4)	116.7(11)	C(13)-C(12)-C(11)	120.39(18)
C(4)-C(5)-C(6)	120.31(18)	C(13)-C(12)-H(12)	119.7(11)
C(4)-C(5)-H(5)	122.4(11)	C(11)-C(12)-H(12)	119.9(11)
C(6)-C(5)-H(5)	117.3(11)	C(14)-C(13)-C(12)	119.71(18)
C(7)-C(6)-C(5)	119.61(19)	C(14)-C(13)-H(13)	120.5(11)
C(7)-C(6)-H(6)	121.1(10)	C(12)-C(13)-H(13)	119.8(11)
C(5)-C(6)-H(6)	119.2(10)	C(13)-C(14)-C(15)	120.15(18)
C(6)-C(7)-C(8)	121.28(17)	C(13)-C(14)-H(14)	121.3(11)
C(6)-C(7)-H(7)	119.4(12)	C(15)-C(14)-H(14)	118.5(11)
C(8)-C(7)-H(7)	119.3(12)	C(14)-C(15)-C(10)	120.56(17)
C(7)-C(8)-N(1)	115.74(14)	C(14)-C(15)-H(15)	118.9(10)
C(7)-C(8)-C(3)	118.83(16)	C(10)-C(15)-H(15)	120.5(10)
N(1)-C(8)-C(3)	125.42(16)	C(1)-N(1)-C(8)	122.15(14)
C(10)-C(9)-S(2)	113.00(13)	C(1)-S(1)-C(2)	102.61(8)
		C(1)-S(2)-C(9)	102.68(9)

Table S10 Anisotropic displacement parameters (Å^2 × 10^3) for **5k**.

The anisotropic displacement factor exponent takes the form: $-2 \pi^2 [h^2 a^{*2} U^{11} + ... + 2 h k a^* b^* U^{12}]$

	U11	U22	U33	U23	U13	U12
C(1)	20(1)	18(1)	14(1)	4(1)	5(1)	3(1)
C(2)	21(1)	21(1)	12(1)	4(1)	3(1)	5(1)
C(3)	19(1)	19(1)	13(1)	5(1)	4(1)	2(1)
C(4)	25(1)	21(1)	16(1)	1(1)	6(1)	0(1)
C(5)	26(1)	24(1)	22(1)	-1(1)	5(1)	-8(1)
C(6)	20(1)	31(1)	24(1)	2(1)	9(1)	-4(1)
C(7)	22(1)	27(1)	19(1)	-1(1)	9(1)	1(1)
C(8)	19(1)	18(1)	13(1)	3(1)	3(1)	1(1)
C(9)	28(1)	18(1)	22(1)	-2(1)	9(1)	0(1)
C(10)	21(1)	18(1)	19(1)	0(1)	9(1)	3(1)
C(11)	26(1)	21(1)	23(1)	-3(1)	7(1)	-2(1)
C(12)	32(1)	23(1)	31(1)	4(1)	14(1)	1(1)
C(13)	28(1)	35(1)	20(1)	6(1)	9(1)	9(1)
C(14)	24(1)	41(1)	22(1)	-8(1)	3(1)	-4(1)
C(15)	27(1)	23(1)	25(1)	-3(1)	10(1)	-5(1)
N(1)	19(1)	21(1)	18(1)	0(1)	6(1)	0(1)
O(1)	25(1)	27(1)	20(1)	-1(1)	9(1)	6(1)
S(1)	20(1)	26(1)	25(1)	-3(1)	11(1)	-2(1)
S(2)	24(1)	23(1)	21(1)	-1(1)	8(1)	-4(1)

Table S11 Hydrogen coordinates ($\times 10^4$) and isotropic displacement parameters ($Å^2 \times 10^3$) for **5k**.

	x	y	z	U(eq)
H(4)	5831(12)	19000(30)	4594(11)	24(5)
H(5)	7257(12)	19220(30)	4417(11)	23(5)
H(6)	7687(14)	16550(30)	3556(12)	27(5)
H(7)	6670(13)	13510(30)	2799(12)	32(5)
H(9A)	4134(13)	6840(30)	1497(11)	29(5)
H(9B)	4922(13)	8870(30)	1861(12)	27(5)
H(11)	4766(12)	12570(30)	943(11)	24(5)
H(12)	4309(13)	14200(30)	-518(11)	31(5)
H(13)	3245(14)	12230(30)	-1713(13)	33(5)
H(14)	2636(14)	8610(30)	-1437(13)	36(6)
H(15)	3031(12)	7030(30)	-23(11)	23(5)

Table S12 Torsion angles [°] for **5k**.

O(1)-C(2)-C(3)-C(4)	0.1(2)
S(1)-C(2)-C(3)-C(4)	-178.77(12)
O(1)-C(2)-C(3)-C(8)	178.67(15)
S(1)-C(2)-C(3)-C(8)	-0.2(2)
C(8)-C(3)-C(4)-C(5)	-1.5(2)
C(2)-C(3)-C(4)-C(5)	177.07(15)
C(3)-C(4)-C(5)-C(6)	0.1(3)
C(4)-C(5)-C(6)-C(7)	1.4(3)
C(5)-C(6)-C(7)-C(8)	-1.4(3)
C(6)-C(7)-C(8)-N(1)	179.57(15)
C(6)-C(7)-C(8)-C(3)	-0.1(3)
C(4)-C(3)-C(8)-C(7)	1.6(2)
C(2)-C(3)-C(8)-C(7)	-176.96(15)
C(4)-C(3)-C(8)-N(1)	-178.10(15)
C(2)-C(3)-C(8)-N(1)	3.4(3)
S(2)-C(9)-C(10)-C(15)	-94.24(18)
S(2)-C(9)-C(10)-C(11)	86.9(2)
C(15)-C(10)-C(11)-C(12)	-0.6(3)
C(9)-C(10)-C(11)-C(12)	178.19(17)
C(10)-C(11)-C(12)-C(13)	-0.6(3)
C(11)-C(12)-C(13)-C(14)	1.3(3)
C(12)-C(13)-C(14)-C(15)	-0.8(3)
C(13)-C(14)-C(15)-C(10)	-0.4(3)
C(11)-C(10)-C(15)-C(14)	1.1(3)
C(9)-C(10)-C(15)-C(14)	-177.70(17)
S(2)-C(1)-N(1)-C(8)	-179.36(11)
S(1)-C(1)-N(1)-C(8)	0.7(2)
C(7)-C(8)-N(1)-C(1)	176.67(15)
C(3)-C(8)-N(1)-C(1)	-3.7(2)
N(1)-C(1)-S(1)-C(2)	1.85(17)
S(2)-C(1)-S(1)-C(2)	-178.13(8)
O(1)-C(2)-S(1)-C(1)	179.08(12)
C(3)-C(2)-S(1)-C(1)	-1.91(14)
N(1)-C(1)-S(2)-C(9)	-5.12(16)
S(1)-C(1)-S(2)-C(9)	174.86(9)
C(10)-C(9)-S(2)-C(1)	-94.06(14)

Fig. S4 HSQC spectrum (500 MHz, 303 K, DMSO-d_6) of 2-(pyrrolidin-1-yl)-4*H*-3,1-benzothiazin-4-one **2c**.

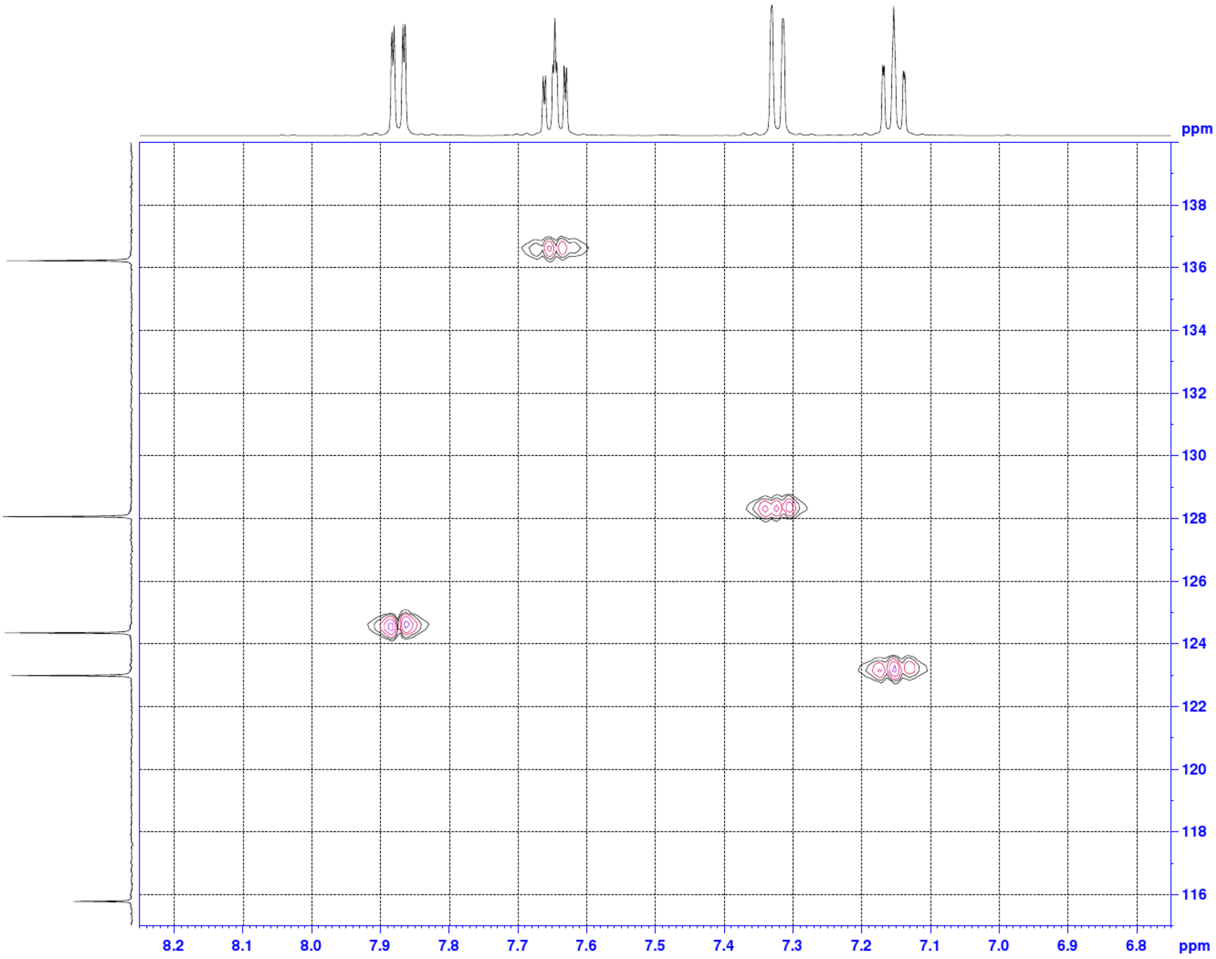

Fig. S5 HMBC spectrum (500 MHz, 303 K, DMSO-d_6) of 2-(pyrrolidin-1-yl)-4*H*-3,1-benzothiazin-4-one **2c**.

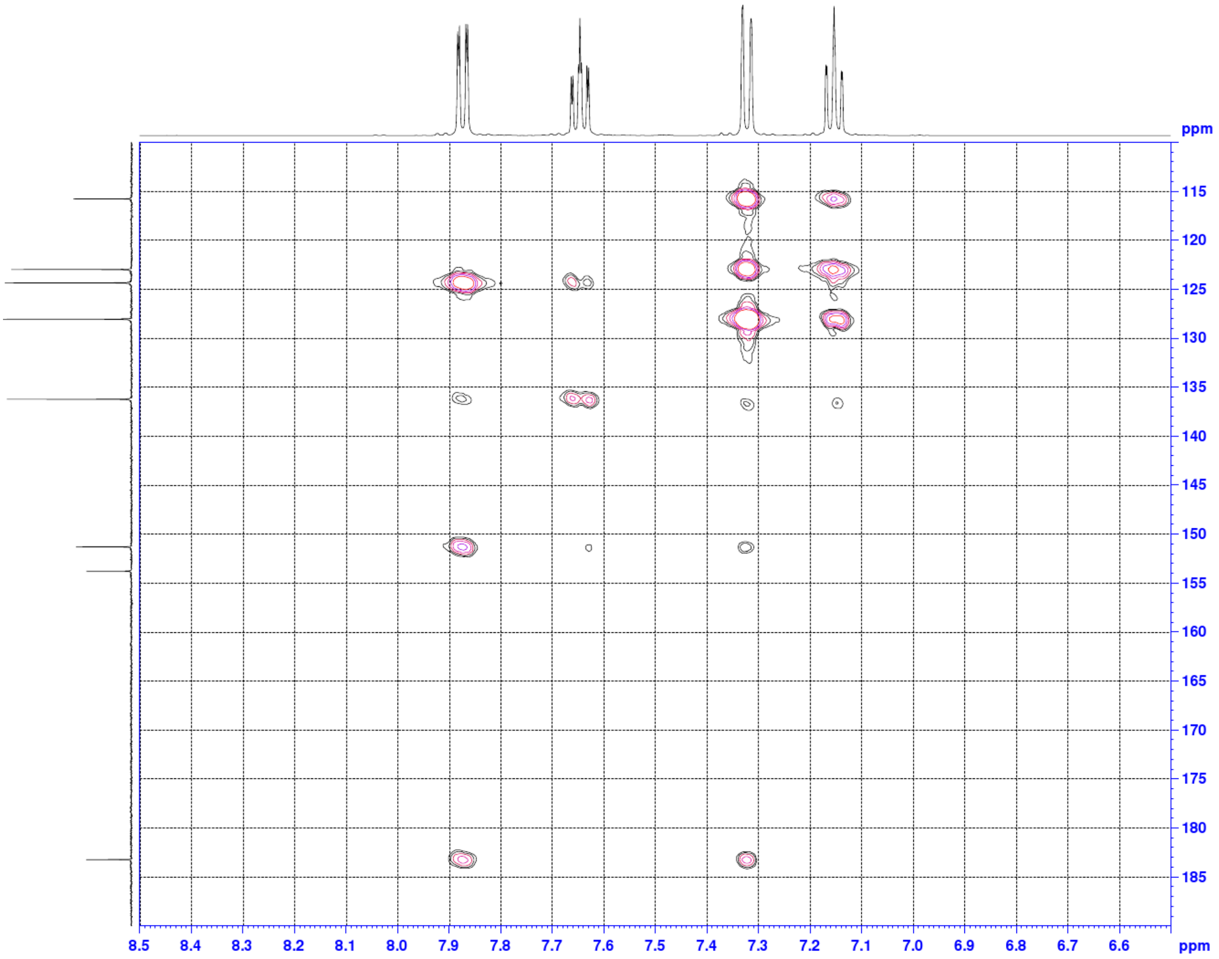

Fig. S6 COSY spectrum (500 MHz, 298 K, $CDCl_3$) of 2-(*N*-benzyl-*N*-methylamino)-4*H*-3,1-benzothiazin-4-one **2g**.

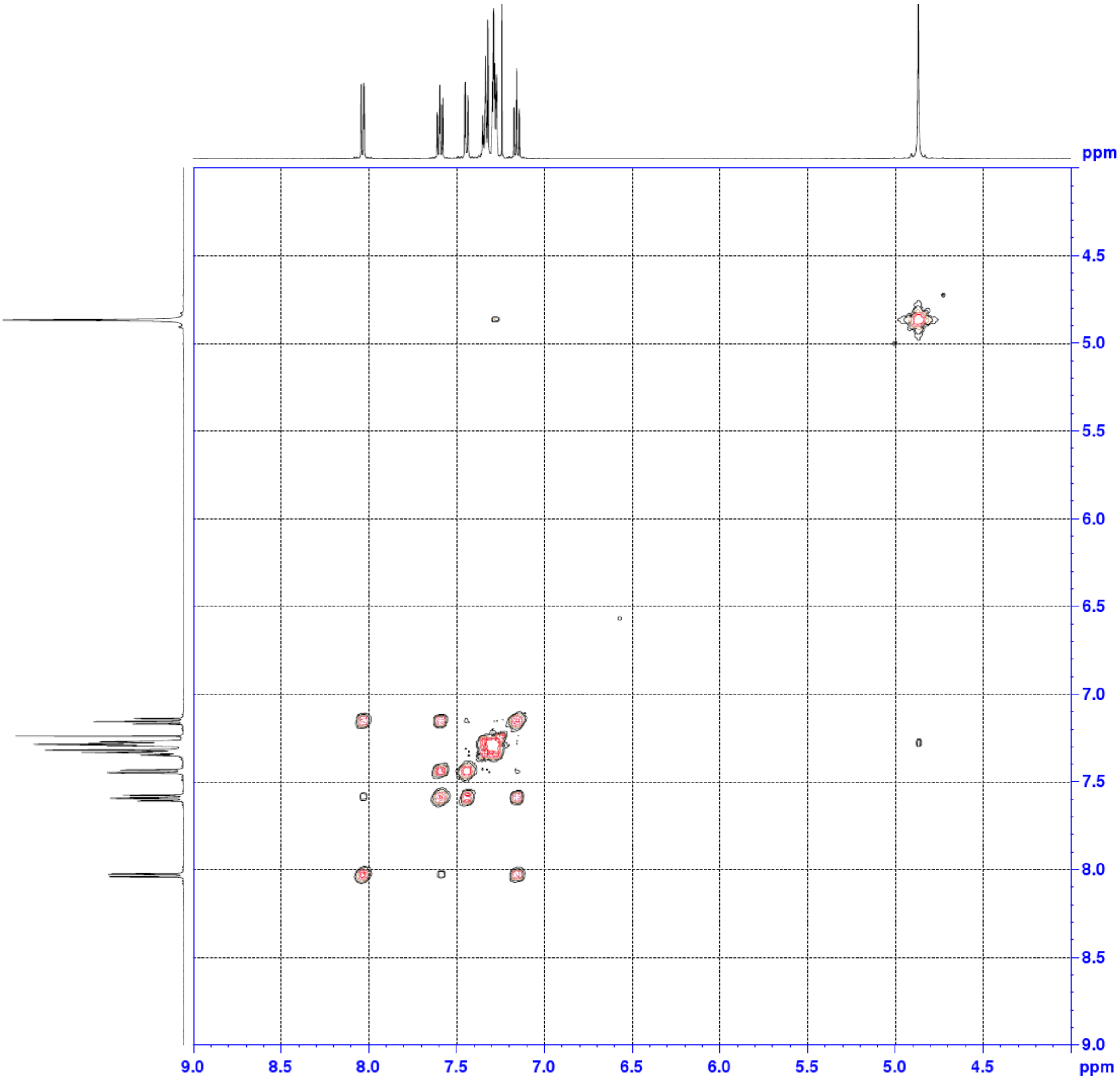

Fig. S7 HSQC spectrum (500 MHz, 298 K, $CDCl_3$) of 2-(*N*-benzyl-*N*-methylamino)-4*H*-3,1-benzothiazin-4-one **2g**.

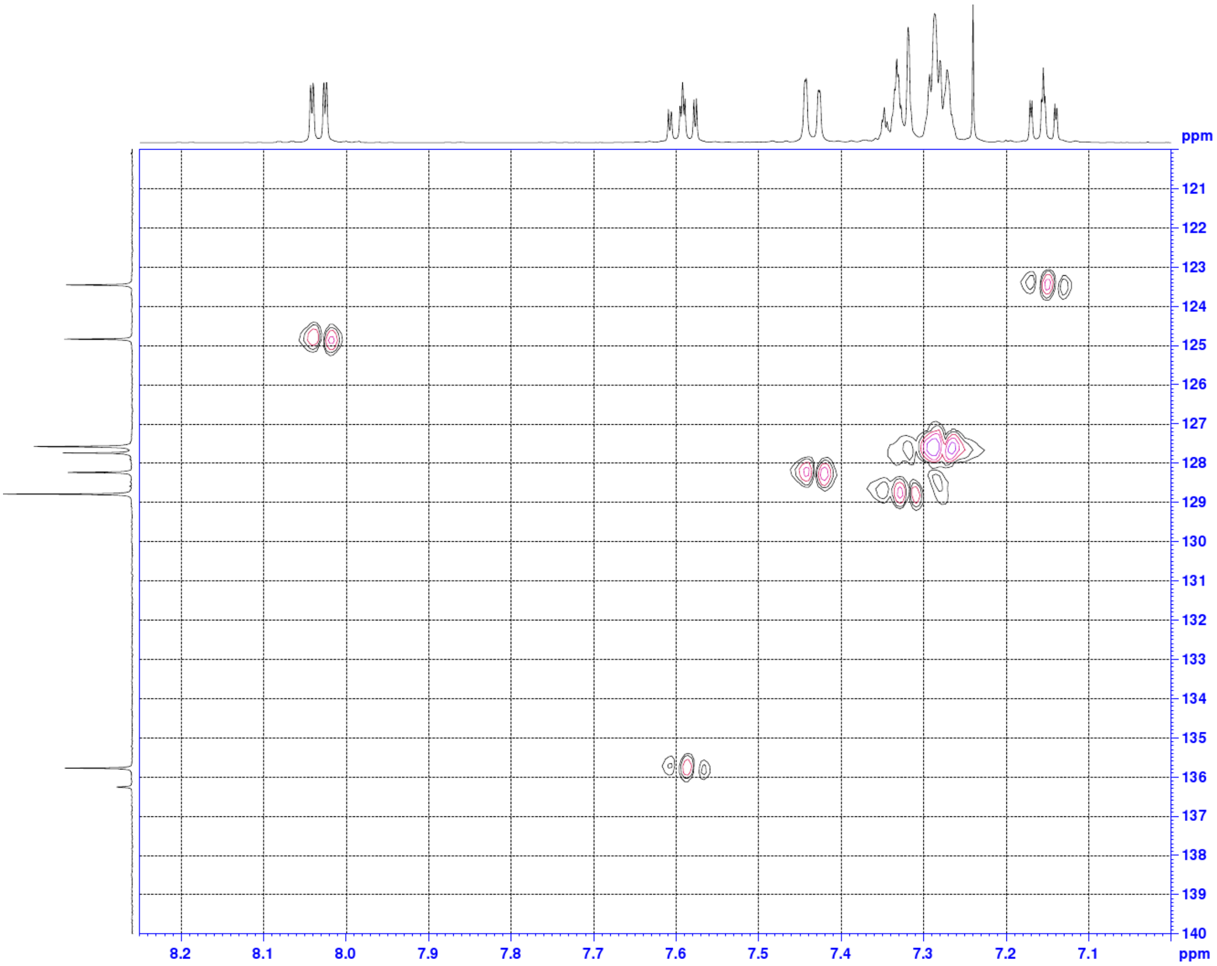

Fig. S8 HMBC spectrum (500 MHz, 298 K, $CDCl_3$) of 2-(*N*-benzyl-*N*-methylamino)-4*H*-3,1-benzothiazin-4-one **2g**.

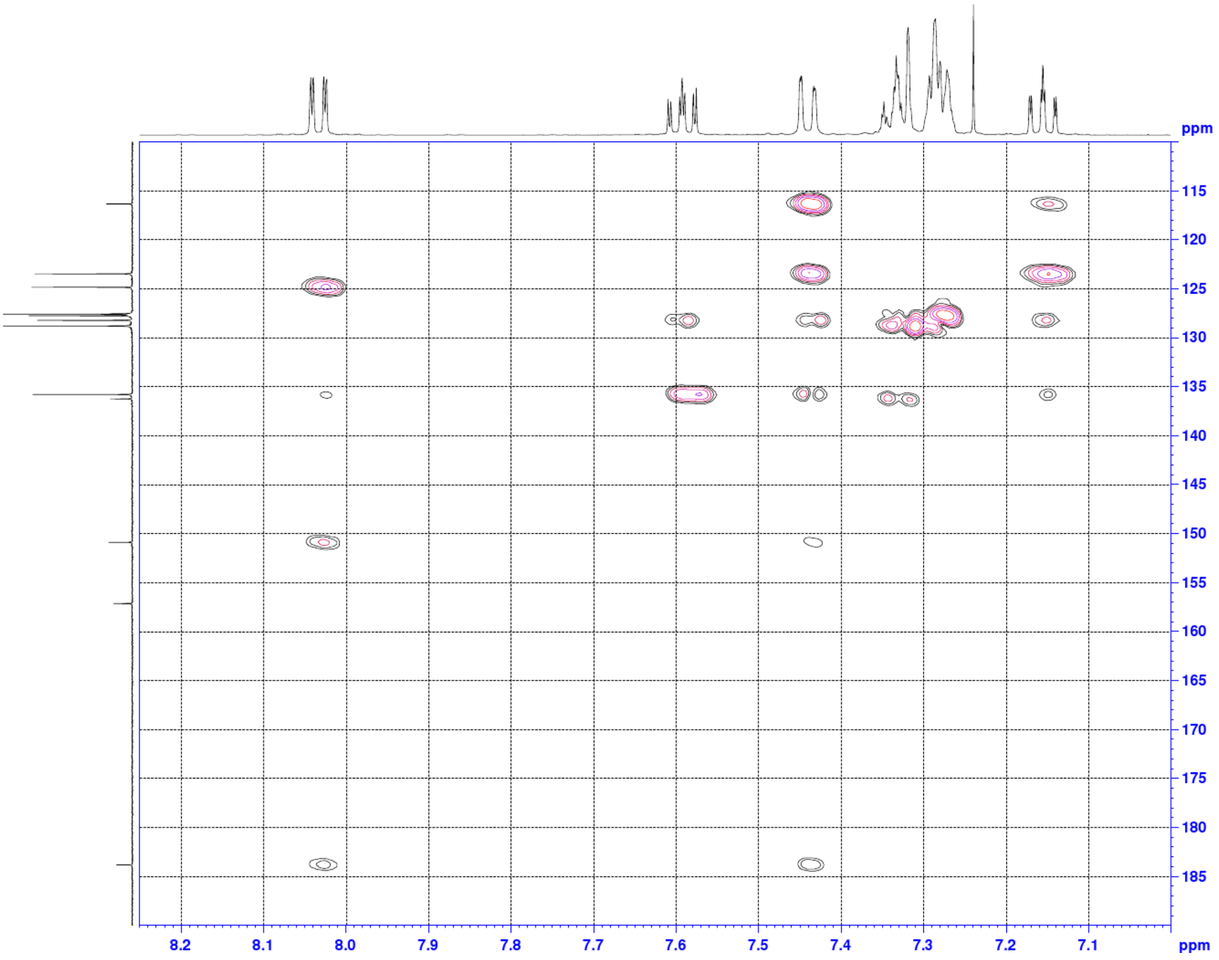

Fig. S9 HSQC spectrum (500 MHz, 298 K, $CDCl_3$) of 2-[(methylthio)thiocarbonylamino]-benzoic acid **4i**.

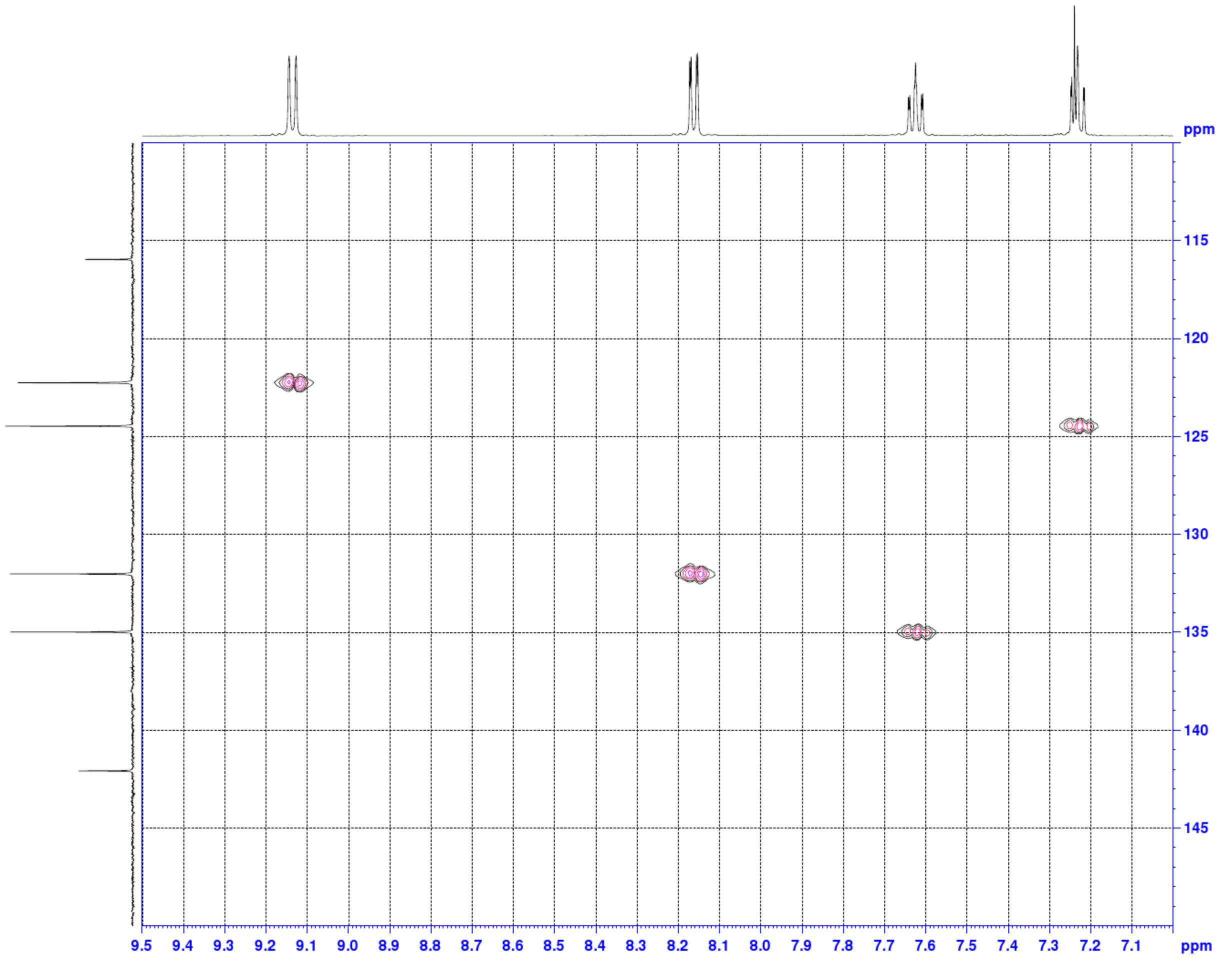

Fig. S10 HMBC spectrum (500 MHz, 298 K, $CDCl_3$) of 2-[(methylthio)thiocarbonylamino]-benzoic acid **4i**.

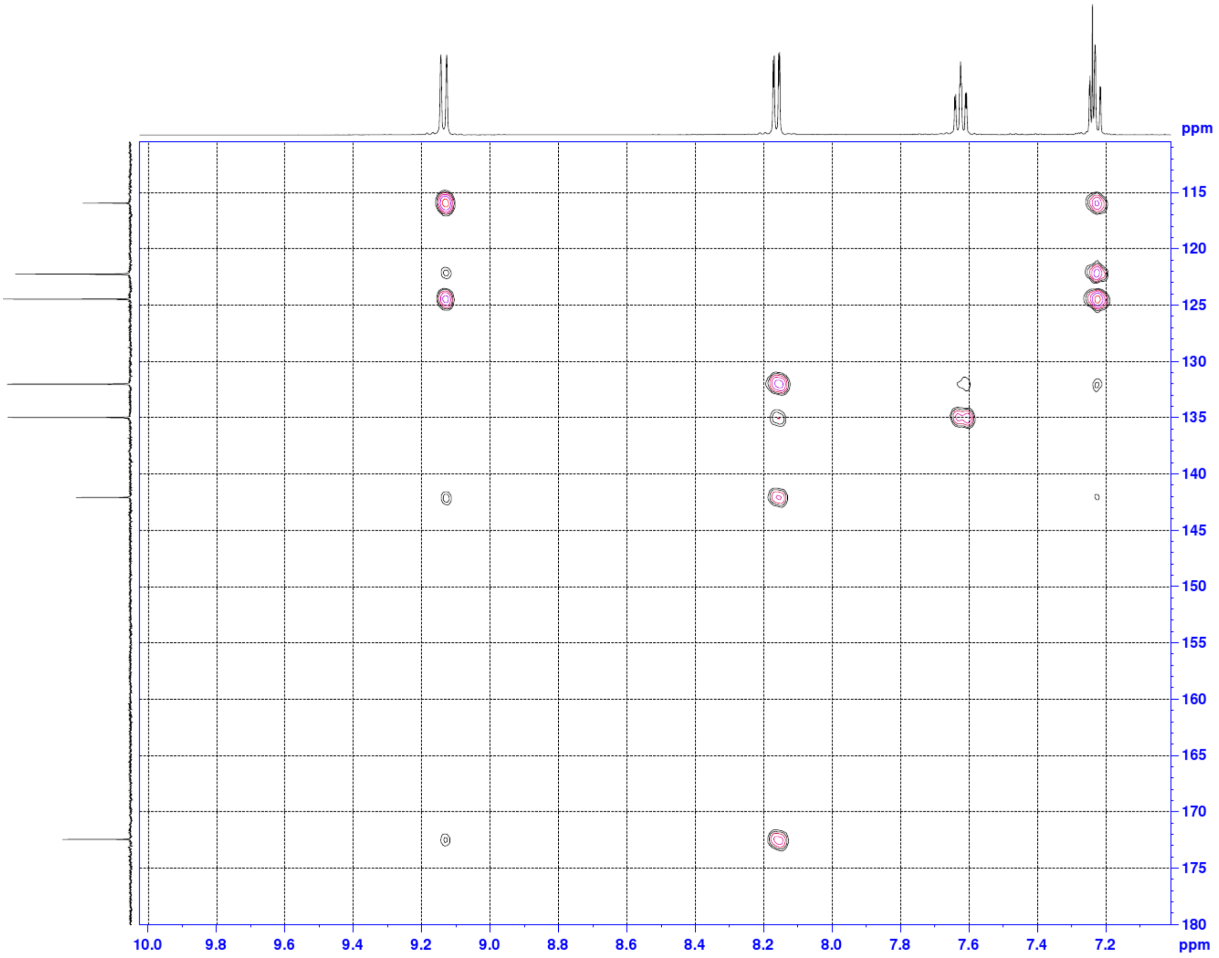

Fig. S11 HSQC spectrum (500 MHz, 303 K, DMSO-d_6) of 2-(methylthio)-4*H*-3,1-benzothiazin-4-one **5i**.

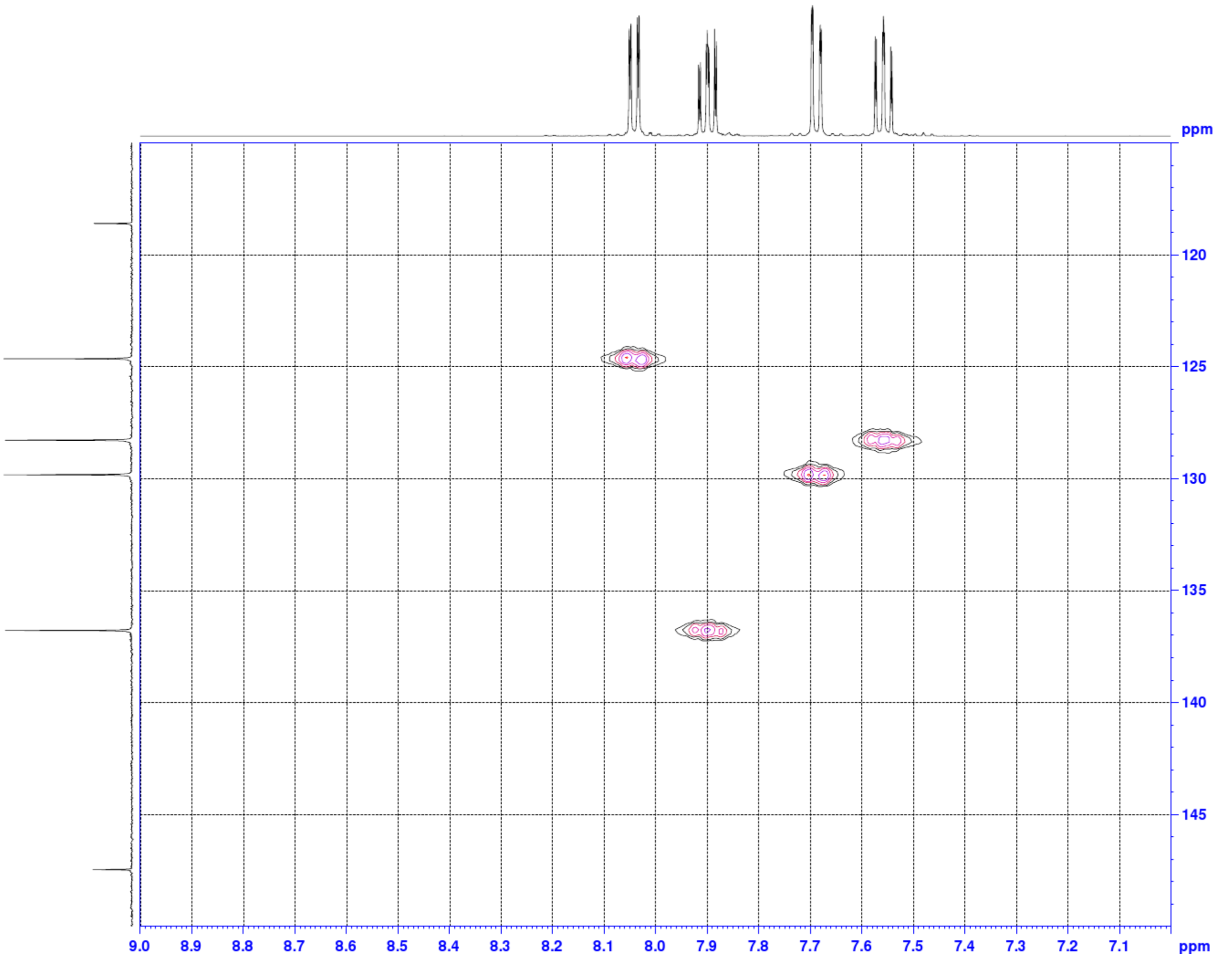

Fig. S12 HMBC spectrum (500 MHz, 303 K, DMSO-d_6) of 2-(methylthio)-4*H*-3,1-benzothiazin-4-one **5i**.

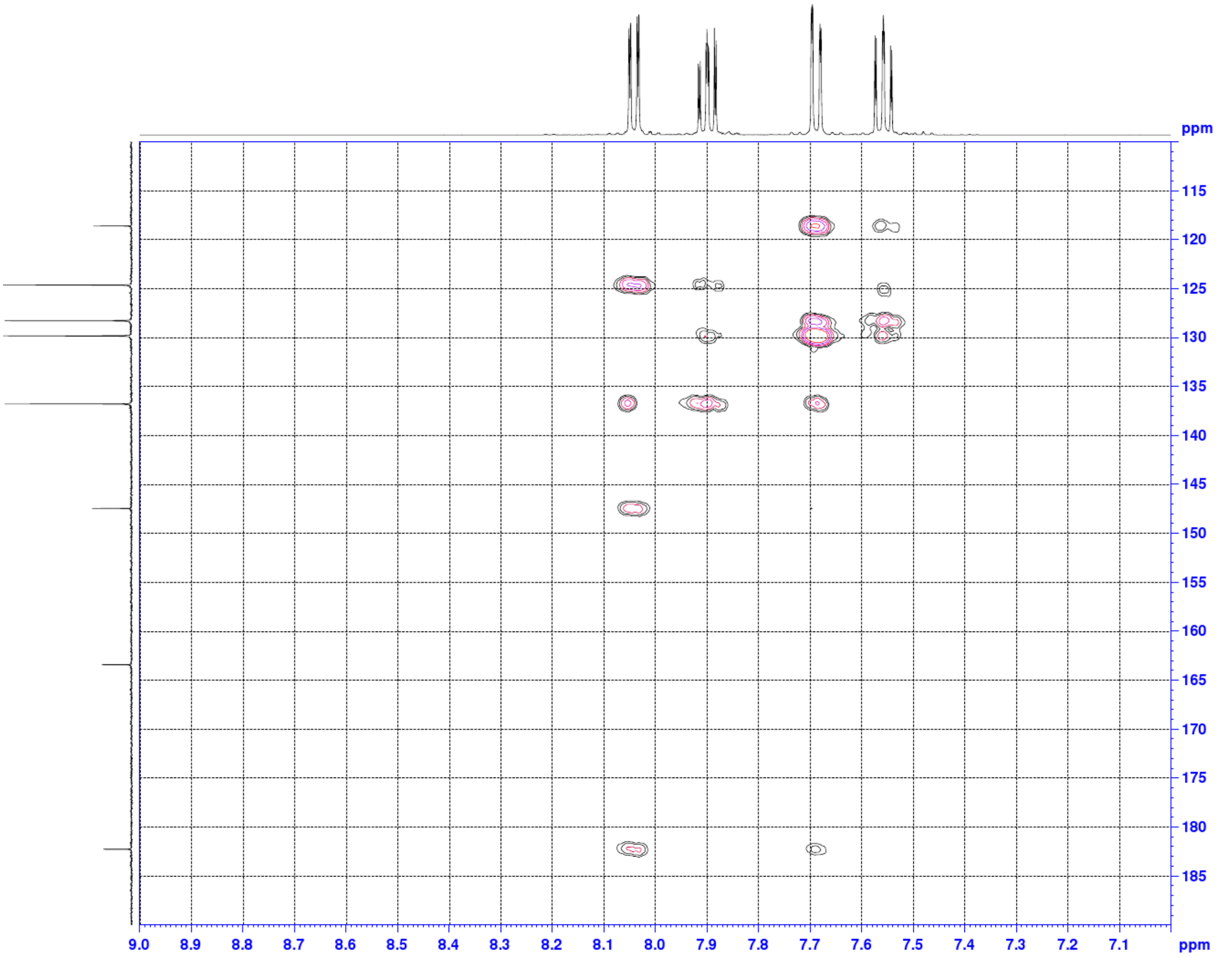

Fig. S13 HSQC spectrum (500 MHz, 303 K, DMSO-d_6) of 2-(3,3-diethylthioureido)-*N*,*N*-diethylbenzamide **6a**.

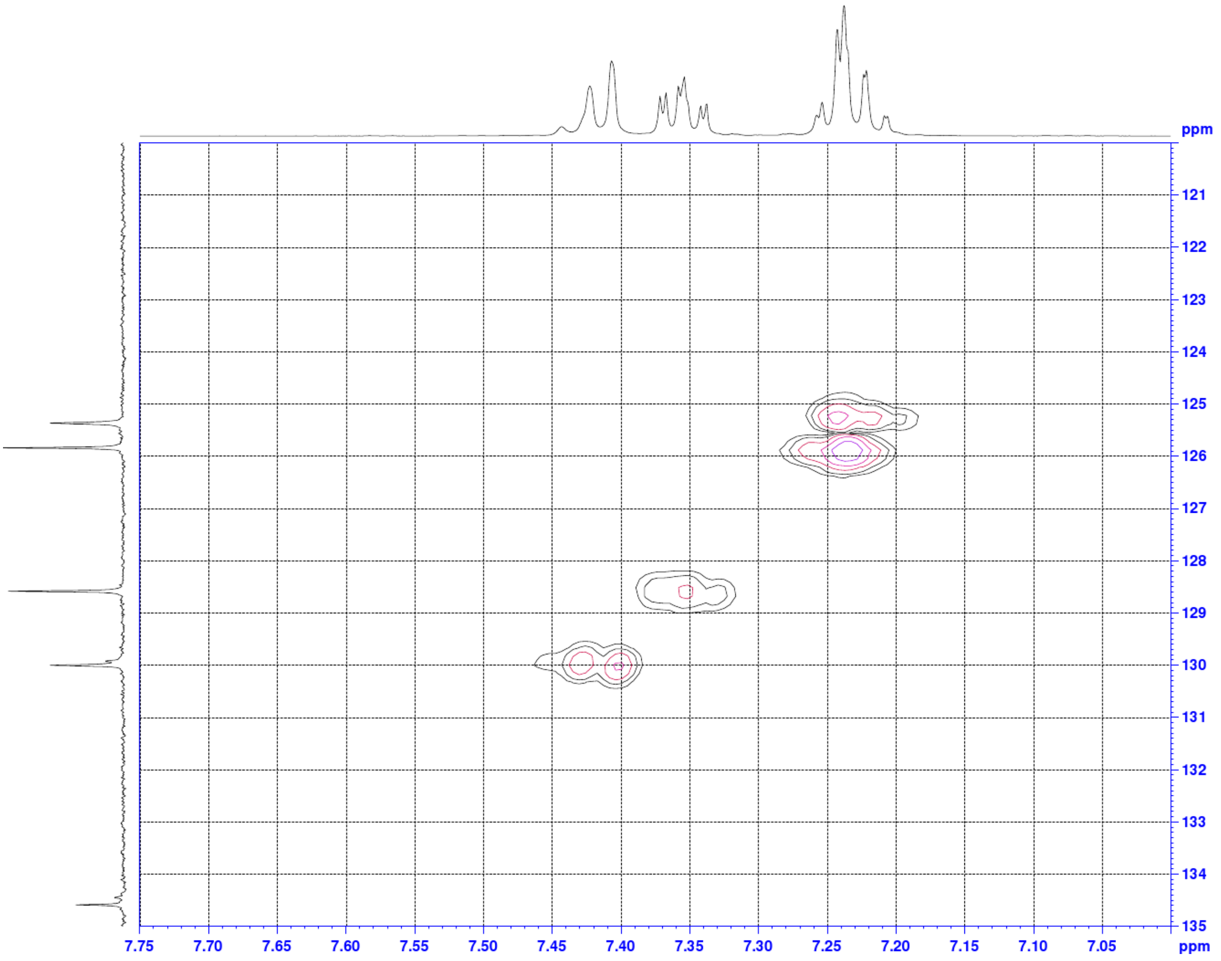

Fig. S14 HSQC spectrum (500 MHz, 303 K, DMSO-d_6) of *N*-[2-(pyrrolidin-1-ylcarbonyl)-phenyl]pyrrolidine-1-carbothioamide **6c**.

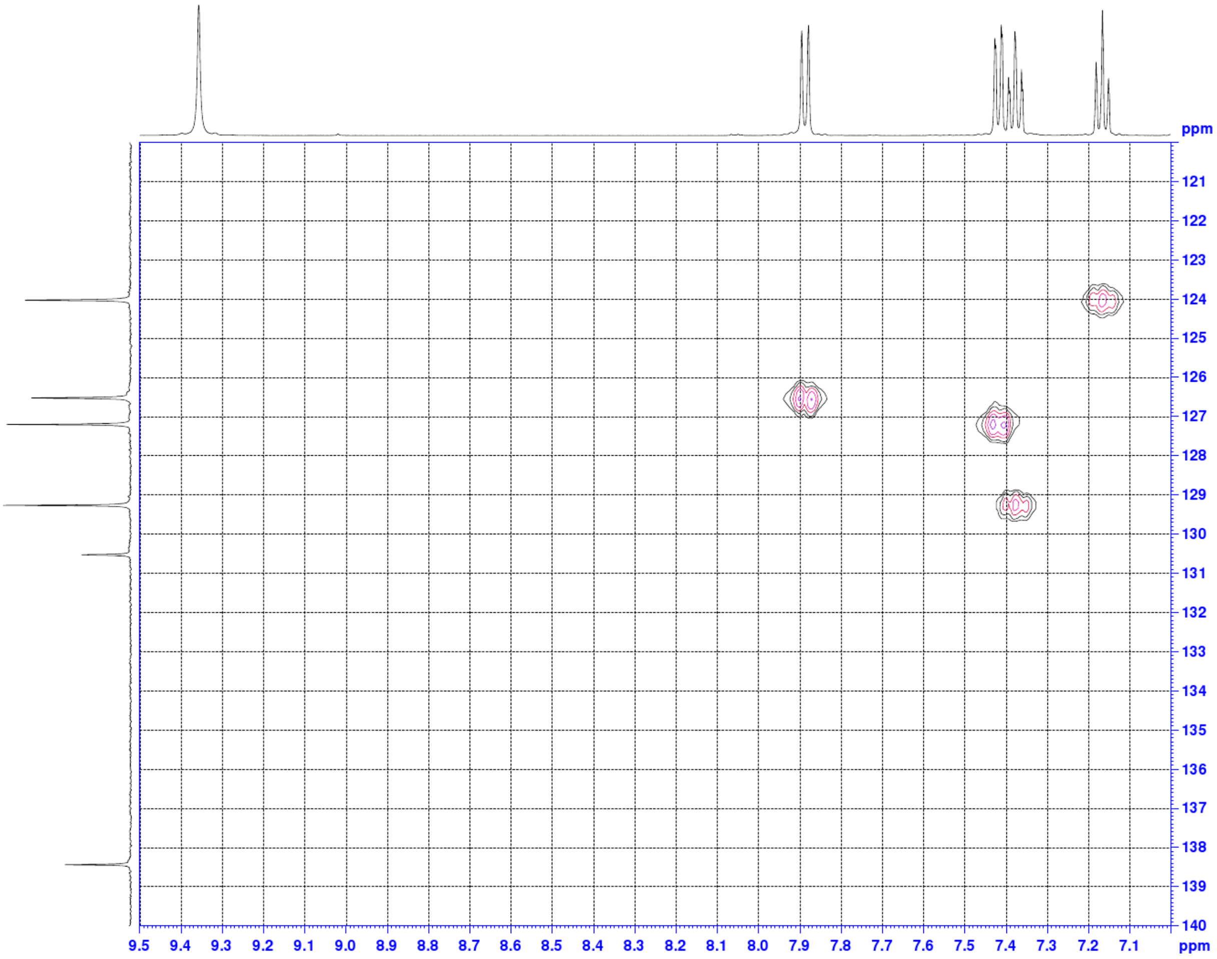

Fig. S15 HSQC spectrum (500 MHz, 303 K, DMSO-d_6) of *N*-[2-(morpholin-4-ylcarbonyl)-phenyl]morpholine-4-carbothioamide **6e**.

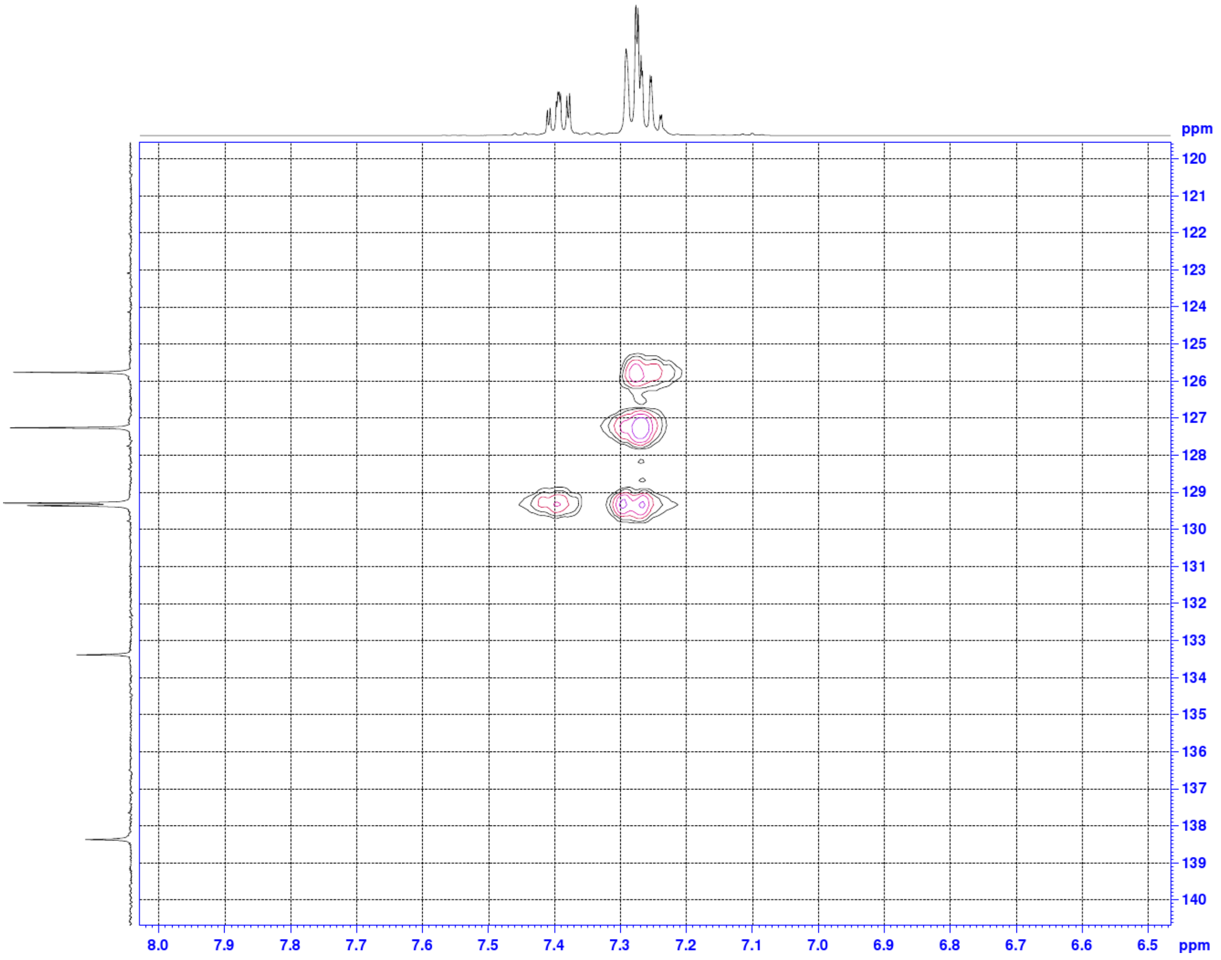

Fig. S16 HMBC spectrum (500 MHz, 303 K, DMSO-d_6) of *N*-[2-(morpholin-4-ylcarbonyl)-phenyl]morpholine-4-carbothioamide **6e**.

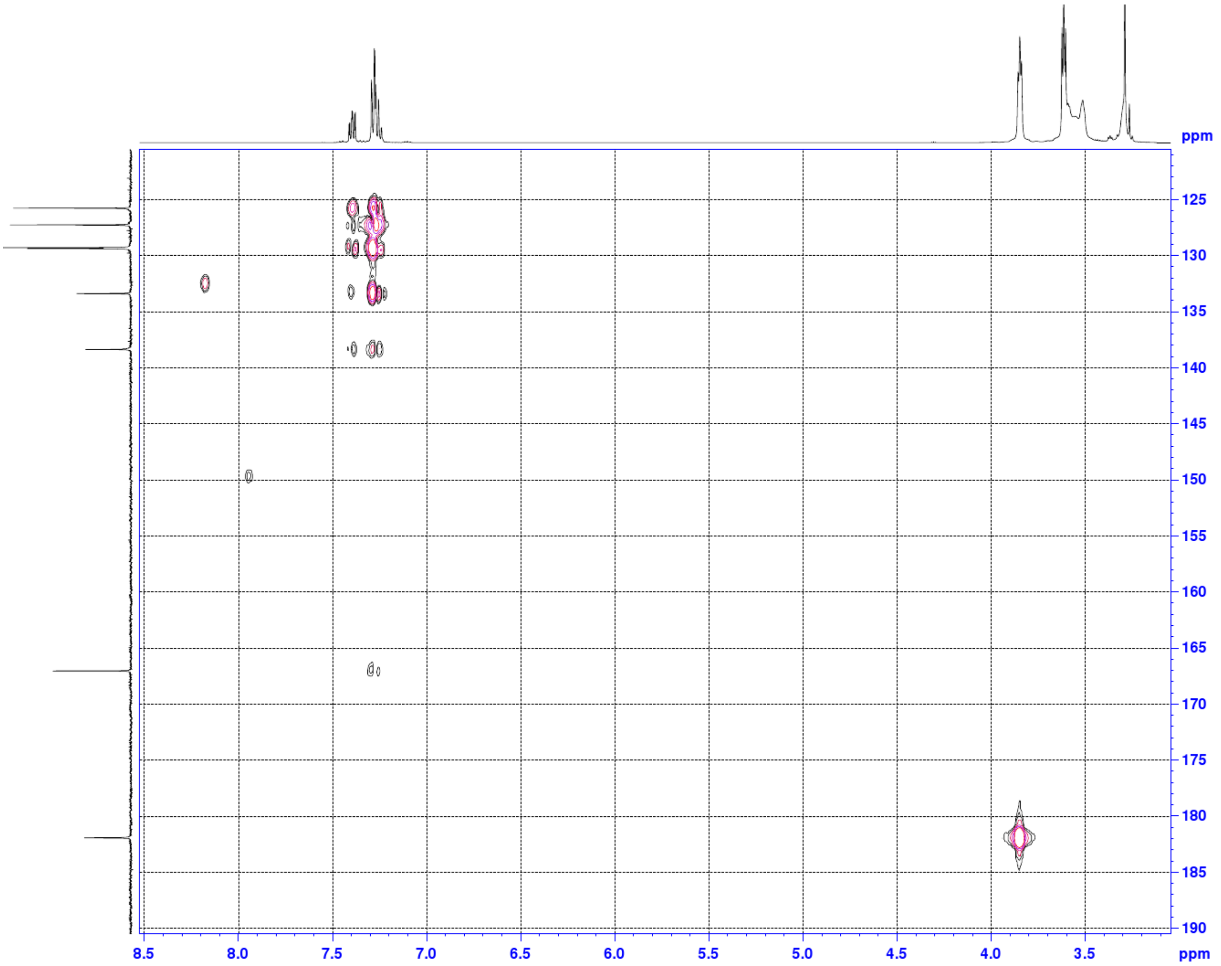

Supplement: Supplementary file 1 [file molecules-14-00378-s001.pdf]
